# Non-Electrode Droplet Manipulation via Triboelectrification Near-Field Energy Transmission

**DOI:** 10.34133/research.1277

**Published:** 2026-05-12

**Authors:** Jun Han, Ruxue Huang, Chuncai Shan, Kaixian Li, Huiyuan Wu, Peng Zhang, Wei Long, Haifeng Qian, Sichen Lu, Bo Pan, Chenguo Hu

**Affiliations:** ^1^ Faculty of Environmental Science and Engineering, Kunming University of Science and Technology, Kunming 650500, P. R. China.; ^2^ School of Physics, Chongqing University, Chongqing 400044, P. R. China.; ^3^ Faculty of Mechanical and Electrical Engineering, Kunming University of Science and Technology, Kunming 650500, P. R. China.

## Abstract

Droplet manipulation technology plays a crucial role in academic research and engineering applications. However, current manipulation technology requires integrated circuits and electrode configurations, which introduce the risk of short circuits and droplet cross-contamination during operation. Herein, we developed an omnidirectional wireless electro-droplet manipulation system (WEDMS) without using electrode/circuit control that obviates droplet cross-contamination to precisely manipulate droplet movement in separated or isolated space. This system innovatively generates localized electric fields through the synergistic effects of dielectric polarization and air discharge, achieving a noncontact, electrode-free droplet charging and driving strategy. It achieves synchronous movement of the slider and droplets via hand movement, enhancing droplet operation flexibility and human–droplet interaction on triboelectric nanogenerators. Moreover, WEDMS is capable of manipulation droplets with high speed across a wide range of volumes (10 to 2,000 μl) and effective control of droplets in both gas and oil phases, achieving a series of manipulations such as droplet positioning, chemical reactions, flexible steering, and droplet climbing, showcasing an innovative, efficient, portable, and comprehensive droplet manipulation strategy. Based on the prominent advantages of WEDMS in structural simplicity and high-efficiency control, its adaptability in multidisciplinary microfluidic applications can be further enhanced, demonstrating tremendous potential in drug delivery, green chemical synthesis, and environmental monitoring.

## Introduction

The manipulation of liquid transport is fundamental to a wide range of biological processes and technological applications [1–3], with significant implications for areas including drug delivery [4,5], environmental monitoring [6,7], biosensing [8,9], and oil–water separation [10]. The efficiency, precision, and versatility of microfluidic and droplet manipulation technologies have driven the development of automated liquid handling systems [11–13]. In modern laboratory practice, liquid samples and reagents require precise handling and transfer, which is typically performed using liquid handling workstations that rely on disposable consumables, including pipettes, centrifuge tubes, and microplates, thereby incurring substantial operational costs. Droplet manipulation methods [14–21] are generally classified as either active or passive, depending on whether external energy input is required, as shown in Fig. S1. Active manipulation employs external stimuli, including electric fields [16], magnetic fields [15], acoustic waves [17], and light-induced forces [14], to actively modulate droplet dynamics. Despite offering rapid response and high spatiotemporal precision, these systems are hindered by bulky power supplies, complex electrode arrangements, and extensive wiring, severely restricting their safety, portability, and integration. Moreover, they may suffer from issues such as droplet contamination, inefficiency in handling large droplets, and evaporation-induced analytical errors. Conversely, passive manipulation techniques exploit the intrinsic properties of materials or physical gradients such as Laplace pressure differences and wettability gradients [20–22] to induce spontaneous droplet motion without the need for external energy. However, these methods rely heavily on predesigned surface structures or gradients, resulting in limited flexibility, weak driving capacity, and susceptibility to uncontrollable environmental factors, which make dynamic, real-time control challenging. Therefore, addressing these challenges is of paramount importance for the advancement of droplet manipulation technology in various application scenarios.

The triboelectric nanogenerator (TENG) was first introduced by Wang’s group in 2012 as a novel energy-harvesting technology based on the coupling of triboelectric electrification and electrostatic induction [23]. It converts low-frequency mechanical energy—including wind, wave energy [24], raindrops, and human motion—into electrical power [25–27]. Therefore, TENGs have established a crucial foundation for the advancement of self-powered sensing devices. [28]. In recent years, TENGs have attracted considerable attention for applications in droplet manipulation [29]. Benefiting from their high-voltage output [30], TENGs can replace conventional high-voltage power supplies for droplet actuation, providing the necessary driving force for droplet manipulation. Zhou et al. developed a rotating TENG to achieve the controllable driving, convergence, and separation of microparticles [8]. Under the influence of dielectrophoresis force, microdroplets move toward the electrodes and coalesce, facilitating lab-on-a-chip applications. Tan et al. [31] developed a noncontact charge injection system by integrating a dual-functional TENG with a bottom circular electrode platform, enabling controllable manipulation of droplets on nonsmooth surfaces. In another study, Sun et al. [19] proposed a droplet manipulation platform based on a self-powered triboelectric tweezer. After rectifying the output signal, charges were directly injected into the droplet through the conductive driving platform. In the linear working mode, an electric field force is generated between the droplet and the tweezer, allowing for precise control of the droplet. Although TENG-based droplet manipulation does not rely on an external power source, TENGs and the droplet manipulation mechanism typically operate as separate units, interconnected via an external rectifier or circuit, with TENGs serving solely as the energy source [18,19,31–33]. This operational mode deviates from the intended design concept of achieving seamless human–machine interaction in droplet manipulation systems. In addition, the associated wiring and electrode connections, which integrate mechanical and electrical components within a confined platform, pose a significant risk of short circuits under conditions of external vibration or operational error. This operation linear mode increases the platform complexity and prolongs the droplet response time. As a result, traditional TENG-based droplet manipulation systems exhibit poor adaptability to complex environments and limited capability for cable-free operation, hindering their broader application. Accordingly, more convenient, flexible, and efficient TENG droplet manipulation technologies are essential to unlock their full potential in human–droplet interaction scenarios.

Herein, we propose a self-powered, cable-free, real-time droplet manipulation strategy by designing an omnidirectional wireless electro-droplet manipulation system (WEDMS). This system does away with the requirement for complex electrode arrays, external high-voltage power supplies, and circuit-based control systems, enabling flexible and precise omnidirectional droplet actuation driven directly by human body movements. The WEDMS platform facilitates omnidirectional friction sliding, simultaneously serving as both the power source and the droplet-driving mechanism. It enables on-demand droplet motion, real-time start–stop control, directional switching, and synchronous response between the slider and droplets. In conventional electrostatic droplet manipulation systems, TENG power generation and droplet actuation modules are independently configured and connected via circuit wiring and rectifiers [19]. This indirect energy transfer mechanism compromises efficiency and incurs considerable energy loss. However, the WEDMS platform combines the TENG power generator and the droplet response platform into an integrated structure, eliminating the need for circuit-based control systems and synchronizing droplet movement during the friction generation. Through the combined effects of dielectric polarization and air discharge, WEDMS establishes a localized electric field capable of real-time, contactless actuation of droplets from 10 to 2,000 μl. WEDMS drives 200-μl droplets at speeds up to 180 mm/s, nearly triple that achieved by conventional electronic control systems. It enables noncontact manipulation of diverse droplets—including inorganic and non-surface-active water-soluble organic solutions and serum along complex paths for applications such as chemical reactions, environmental sensing, and solid–liquid mixing. This strategy markedly improves system integration, operational simplicity, and the adaptability of human–droplet interfaces.

## Results and Discussion

### Design and mechanism of WEDMS wireless human–droplet interaction platform

**Fig. 1.**
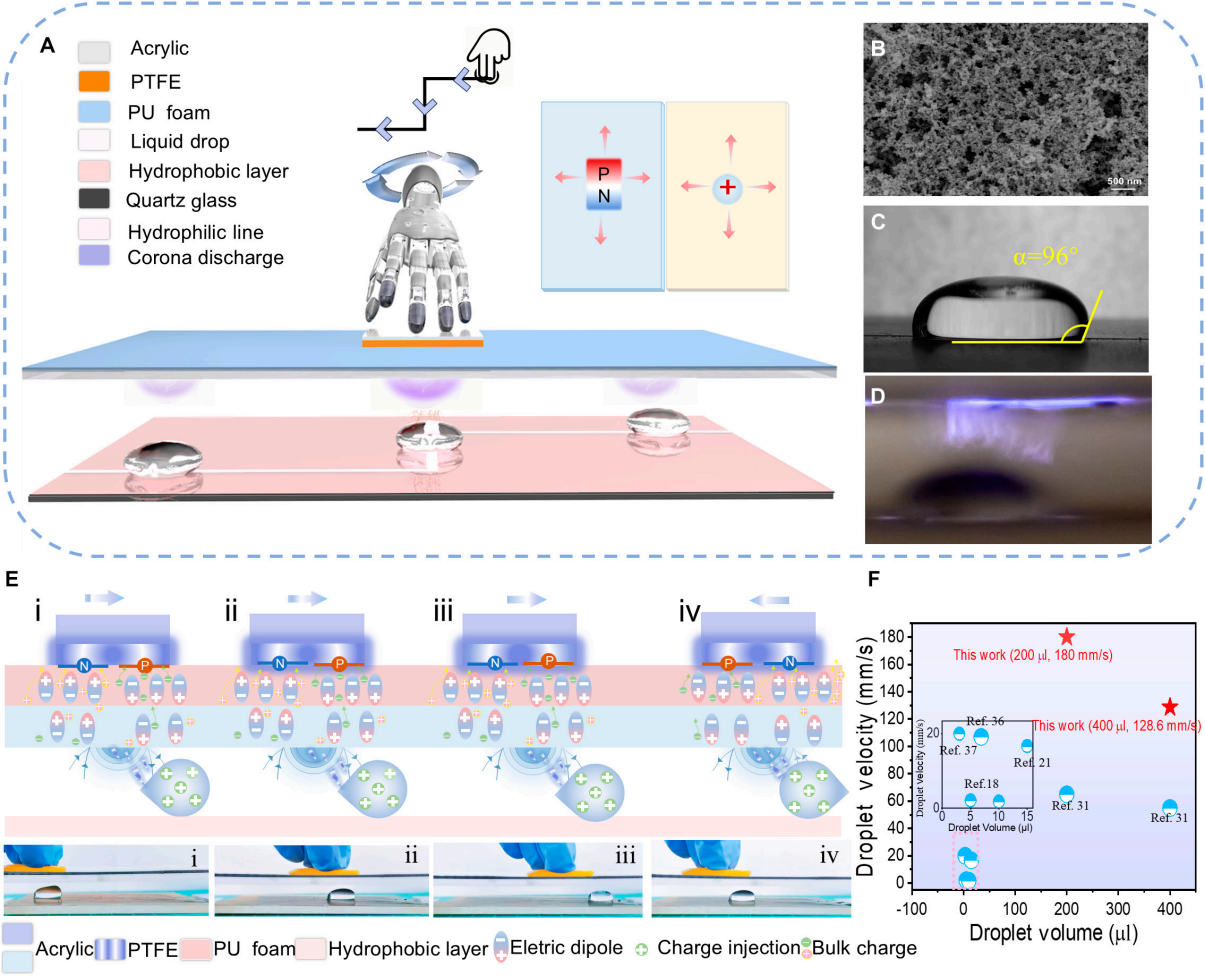
WEDMS system for multimodal droplet manipulation. (A) 3D schematic diagram based on WEDMS wireless human–droplet interaction platform. (B) SEM image depicting the NC319 hydrophobic layer. (C) Measurement of the contact angle for an 800-μl droplet on the hydrophobic layer with hydrophilic lines. (D) DRP-TENG discharge photo taken by a high-speed camera. (E) Working principles of the DRP-TENG contactless actuation of droplets. (F) Comparison of the moving velocities of droplets with various manipulation methods.

The WEDMS droplet manipulation method overcomes the limitations of traditional systems by eliminating external high-voltage power supplies and avoiding complex circuits that are prone to short-circuiting, offering a simple, efficient, and widely applicable approach. As shown in Fig. 1A, the WEDMS platform integrates a TENG and a droplet manipulation module. The TENG’s slider serves as both the energy source and the actuator, directly coupling with the droplet manipulation unit to control droplet motion. It can be observed that there is a certain spacing between the upper and lower platforms, which facilitates the provision of path space for air discharge between TENG and liquid droplets. The complex manipulation of liquid droplets is achieved through mechanical hand motions, where interlinked upper and lower platforms couple triboelectric fields with droplet control, facilitating direct human–droplet interaction. The experimental device, depicted in Fig. S2, includes a TENG-hand control platform and a droplet motion platform, and the WEDMS system efficiently maneuvered the droplets along the curve, as shown in Movie S1. In this system, the upper platform features a dual-region opposite-polarization TENG (DRP-TENG) [34,35]. Polytetrafluoroethylene (PTFE) film and polyurethane (PU) foam, with a significant difference in triboelectric polarity, are used as the triboelectric materials. The triboelectric layer is mounted on an acrylic plate, where PU acts both as a triboelectric material and as a buffer layer, ensuring close contact between the friction components (Note S1). Additionally, the PTFE surface is smooth and flat and the PU surface shows uniform microporosity (Fig. S3). DRP-TENG efficiently converts human body sliding motion into electrical energy, enabling droplet manipulation while facilitating human–droplet interaction and expanding human–machine interfaces. The lower droplet-driving platform consists of quartz glass covered with a hydrophobic coating, in which a micrometer-scale hydrophilic line is patterned at the center to direct droplet movement. The scanning electron microscopy (SEM) image of the Narroco NC319 hydrophobic layer is shown in Fig. 1B. Compared to the droplet contact angle on the NC319 hydrophobic material, depicted in Fig. S4, the contact angle of a droplet of 800 μl located on the hydrophobic layer with hydrophilic lines in air is 96° (Fig. 1C) and the surface of the NC319 hydrophobic layer contains micrometer-scale hydrophilic lines, which reduce the contact angle of droplets to some extent. In the field of hydrophobic materials, the contact angle is a key parameter for evaluating droplet-driving performance. A larger contact angle typically correlates with the lower frictional resistance, making it easier to drive the droplet. The DRP-TENG devices equipped with this hydrophobic layer with hydrophilic lines can still drive droplets smoothly and efficiently, even at a contact angle of 96°. This fully demonstrates the strong adaptability and excellent driving performance of TENG devices under complex interface conditions. Meanwhile, it also highlights the advantages of the NC319 hydrophobic layer with hydrophilic lines when working in concert with TENG. Additionally, a purple glow discharge induced by air breakdown in the gap between TENG and the droplet was captured using a digital camera with a 30-s exposure (Fig. 1D). This glow emission reveals the discharge path at the air gap during DRP-TENG operation. The corona discharge generates a strong localized electric field, forming a transient conductive pathway that injects charge into the droplet and enables its controlled, noncontact actuation. Furthermore, the output performance of corona discharge is also influenced by the surrounding environment, particularly relative humidity. Studies have shown that the output charge of the DRP-TENG triboelectric interface corona discharge decreases with increasing relative humidity (Fig. S5). The long-term operational stability of WEDMS is shown in Fig. S6.

The working principles of DRP-TENG are illustrated in Fig. 1E. This system enables real-time, noncontact droplet actuation based on 2 key mechanisms: (a) The large electronegativity difference between PU foam and PTFE produces a strong electric field via contact electrification, which injects charge into the droplet (as illustrated in Note S2) and generates a high coulomb force to drive its motion. (b) As the slider moves across the PU surface, the dielectric layers at the front and rear of the slider exhibit opposite polarization field distributions, accompanied by a dynamic polarization process. This dynamic and reversible polarization enables the formation of controllable, direction-dependent electric fields, allowing the droplet to start, stop, as shown in Movie S2, and change direction in synchrony with the slider’s motion. Previous studies have shown that electrodes placed on both sides of a PTFE slider produce opposite discharge paths relative to the PTFE and PU layers, and it is therefore hypothesized that a similar discharge pattern exists directly beneath the slider. To test this hypothesis, 2 electrodes are placed in the front and rear regions below the slider (with the slider electrode removed) and are moved dynamically with the slider [35], as shown in Fig. S7. The results indicate that when the slider moves from left to right, the direction of charge transfer at the corresponding front and rear electrodes is opposite, and the output current remains constant. In the initial stage [Fig. 1E(i)], the electrodes are replaced by droplets, which are positioned in the area corresponding to the front of the slider. Due to the electrodeless configuration of TENG, a strong localized electric field forms on the tribo-layer interface, resulting in the polarization of the dielectric polymer and the formation of a quasi-dipole potential distribution. The induced electric dipole at the bottom of the substrate can be regarded as bound surface charges, with the left side of the PTFE triboelectric layer exhibiting a negative potential and the right side a positive potential. When the electric field intensity exceeds the air breakdown threshold, continuous corona discharge occurs on the right side of the slider, continuously injecting positive charges into the target droplet. The positively charged droplet experiences a coulomb repulsive force from the positive electric field on the left, driving it rightward synchronously with the slider until the operation step is completed [Fig. 1E(i to iii)]. When the droplet completes its unidirectional motion and the slider reverses from right to left, the interfacial potential distribution switches accordingly, with the PTFE layer becoming positively charged on the left and negatively charged on the right [Fig. 1E(iv)]. Positive charges are injected into the droplet at its leading edge, while the negatively charged region of the slider attracts the droplet leftward, illustrated in Fig. S8 and Movie S3 demonstrating the operational principle of DRP-TENG, while a comprehensive analysis is provided in Note S3. Subsequently, when we suddenly stop actuating, observing that with the removal of the applied electric field, the corona discharge cannot be sustained and quickly extinguishes. The charge transfer and injection process between the triboelectric layer interface and the droplet immediately cease, as shown in Fig. S9. Benefiting from this design, WEDMS enables the reliable actuation of droplets ranging from 10 to 2,000 μl, achieving velocities of up to 180 and 128 mm/s for 200- and 400-μl droplets, respectively (Fig. 1F). Compared to conventional electronically controlled systems [18,21,31,36,37], the WEDMS platform demonstrates clear advantages in both droplet volume capacity and actuation speed, further highlighting its stability, controllability, and practical applicability.

### Electrostatic field modulation and droplet transport characteristics

**Fig. 2.**
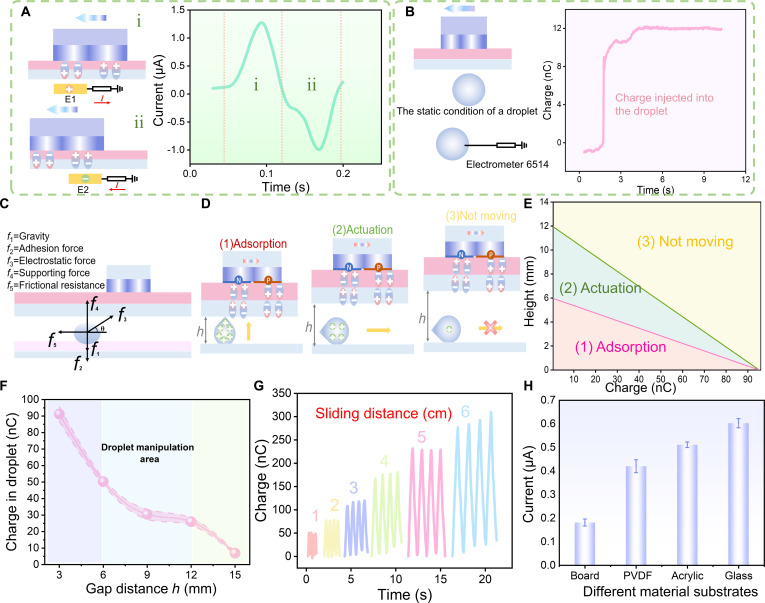
Electrostatic field regulation and characteristics of droplet transportation. (A) Schematic diagram of verifying the quasi-dipole potential distribution at the dielectric polymer interface during its movement and the current output of the structure. Labels (i) and (ii) depict slider regions entering electrodes E1, E2, with corresponding current signals. (B) Schematic diagram of the charge injection mechanism into the droplet and its charge output characteristics. (C) Force analysis of droplets in a WEDMS system. (D) Schematic diagram of the droplet dynamic behaviors by adjusting the height difference between TENG and the droplet. (E) The effective actuation range of the WEDMS system is determined by the relationship between the charge amount and the height difference. (F) Relationship between the different gap distance ℎ and the charge injected by the driving droplet. (G) Relationship between the different distances of the slider slides and the charge output of TENG. (H) Current output characteristics of TENG with different substrate materials.

To verify the effect of the slider on the quasi-dipole potential distribution at the dielectric polymer interface during its movement, static electrodes are placed beneath the polymer substrate [34,35]. The corona discharge currents are measured in real-time as the left and right parts of the slider moved into the electrode region in Fig. 2A. The experimental results indicate that when the PTFE slider moves from right to left, the interface potential corresponding to the left half of the slider becomes positive. This drives the quasi-dipoles at the friction interface to align along the electric field direction (from top to bottom) and induces a positive discharge signal. In contrast, when the right half of the slider moves into the region directly above the electrode, the interface potential becomes negative, inducing a negative corona discharge toward the electrode and generating a corresponding negative current signal. The current curve in Fig. 2A shows an initial positive followed by a negative signal, consistent with the expected quasi-dipole potential distribution, confirming the model’s validity and the slider’s effective control over dielectric polarization (Movie S4). Additionally, we measured the impact of the opposing potentials generated at both ends of the PTFE slider during sliding on the quantity of charge injected into the droplets. As shown in Fig. 2B, the left panel illustrates the charge injection mechanism into the droplet, while the right panel shows the corresponding output. Initially, the droplet is placed on the lower surface of TENG, 9 mm away. The front half of the slider then moves across the droplet, with no electrode within the electric field range during this phase. Subsequently, the electrometer electrode contacts the droplet and registers a positive charge injection (during this process, the slider remains stationary). The droplet is injected with 12-nC charge. Control tests confirmed that no charge injection occurred when only the negatively charged region passed over the droplet, leaving its net charge nearly zero. When the gap exceeds 12 mm, ensuring that the droplet remains stationary, the charge carried by the droplet continuously accumulates as the PTFE slider completes a reciprocating motion above it. When the negatively charged end of the slider passes near the droplet, the positively charged droplet exhibits a higher potential than the surrounding negative potential. As a result, the slider does not inject negative charge into the droplet; instead, it exerts an attractive force on the positively charged droplet, as shown in Fig. S10.

WEDMS primarily employs electric field force to actuate the droplet and analyzes the forces acting on the driven droplet (Fig. 2C). The droplet is subjected to 5 primary forces: gravity (*f*_1_), adhesion force (*f*_2_) on a 3-phase hydrophilic line, electrostatic force (*f*_3_) , supporting force (*f*_4_), and frictional resistance (*f*_5_).

$$f_{1}=\mathrm{mg}=\rho_{0}\mathrm{Vg}$$ (1)

$$f_{2}=2\pi r_{0}\gamma \sin\theta$$ (2)

$$f_{3}=k\frac{q_{1}\times q_{2}}{h^{2}}$$ (3)

$$f_{4}=f_{3}\sin\theta -f_{1}-f_{2}$$ (4)

$$f_{5}=w\gamma_{1}k_{1}\left(\cos\theta_{r}\;-\;\cos\theta_{\alpha }\right)$$ (5)

where *g* represents the gravitational acceleration, *V* denotes the droplet volume, $\rho_{0}$ refers to the droplet density ($\rho_{0}=$ 1 g/cm^3^), *r*_0_ is the droplet contact radius, *γ* is the surface tension (γ = 72.8 mN/m), *θ* is the contact angle, *k* represents the coulomb constant (*k* = 9 × 10^9^ N·m^2^/C^2^), *q*_1_ is the charge on WEDMS, *q*_2_ is the charge within the droplet, *h* is the distance between the PU substrate and the droplet, *w* denotes hydrophilic line width (*w* = 0.1 mm), $\gamma_{1}=\frac{G}{V},$ *k*_1_ is Droplet shape correction factor, and *θ_r_* and *θ_α_* are the backward contact angle and forward contact angle, respectively. The droplet’s movement is governed by the electric field generated by the lower surface of TENG and the charge injected into the droplet. The electrostatic force acting on the droplet is dependent on the distance *h* between the triboelectric layer and the droplet’s driving platform. The distance between the triboelectric layer and the droplet must fall within the effective driving distance *h*_2_ (where *h*_1_ < *h*_2_ < *h*_3_) in Fig. 2D. COMSOL software is employed to simulate the static electric field distribution of DRP-TENG and the droplet assembly in 3 different distances (Fig. S11). Through the adjustable electrostatic interaction between the droplet and the friction substrate, we have identified 3 distinct dynamic behaviors of the droplet via theoretical analysis and experimental trials. As shown in Fig. 2E and the process depicted in Movie S5, in region (1), excessively small spacing causes excessive charge injection into the droplet, increasing the electrostatic force and resulting in its adhesion to TENG, thus causing driving failure. In region (3), if the spacing is too large, air discharge occurs at the bottom surface of TENG, preventing effective charge injection and leading to charge loss. In this case, the horizontal electrostatic force becomes insufficient to overcome frictional resistance, and the droplet remains stationary. The effective gap range for the droplet and quartz substrate is 6 to 12 mm (region 2). Beyond this, actuation fails due to electrostatic force limitations, restricting motion to the area directly beneath the slider. We also observed that within the effective driving spacing, droplets undergo slight and unavoidable deformation under the influence of the electric field. However, the overall interfacial forces remain balanced, and the droplet structure is not compromised. Upon cessation of the driving force, the droplets rapidly return to their initial shape.

The effect of DRP-TENG (WEDMS) spacing and substrate composition on droplet actuation is investigated. As shown in Fig. 2F, increasing the gap distance *h* from 3 to 15 mm reduces the discharge transfer charge collected by a fixed electrode at the droplet position, following the same trend as the current and the voltage (Fig. S12). Figure 2G shows the output charge characteristics of DRP-TENG at a sliding speed of 0.5 m/s for different sliding distances. The results indicate that, under constant conditions for other parameters, the transferred charge is proportional to the sliding distance, with the current stabilizing around 0.6 μA. As the sliding distance increases, the accumulated charge at the interface continues to rise, gradually increasing the interface voltage (Fig. S13). In accordance with Paschen’s law:

Vb=APdlnPd+B (6)

Here, *V_b_* denotes the voltage at which breakdown occurs, *A* and *B* are constants determined by the gas composition and gas pressure (in ambient air at room temperature, *A* = 273.75 (V/Pa·m), *B* = 1.08), *d* represents the gap distance between the 2 layers, and *P* refers to the gas pressure. The breakdown voltage experimentally measured in this system is 8,237 V (Note S4). With increasing gap distance from electrode and the electric field, corona discharge becomes less likely, potentially ceasing altogether, which leads to a decline in output performance.

Furthermore, we also examined the influence of the triboelectric layer substrate on the charge transport by selecting 4 different substrates—polyvinylidene difluoride (PVDF), acrylic, wood, and quartz—and measuring the current of a 1-ml droplet at a spacing of 9 mm. The current is the lowest under the wood substrate and the highest under the quartz substrate, and the current under PVDF and acrylic substrates is approximately equivalent (Fig. 2H). This variation is attributed to the dielectric and polarization properties of the substrates. The amount of charge and voltage output performance of the injected droplets under different substrates shows the same trend as the current (Fig. S14).

### High-speed cable-free droplet manipulation via WEDMS

**Fig. 3.**
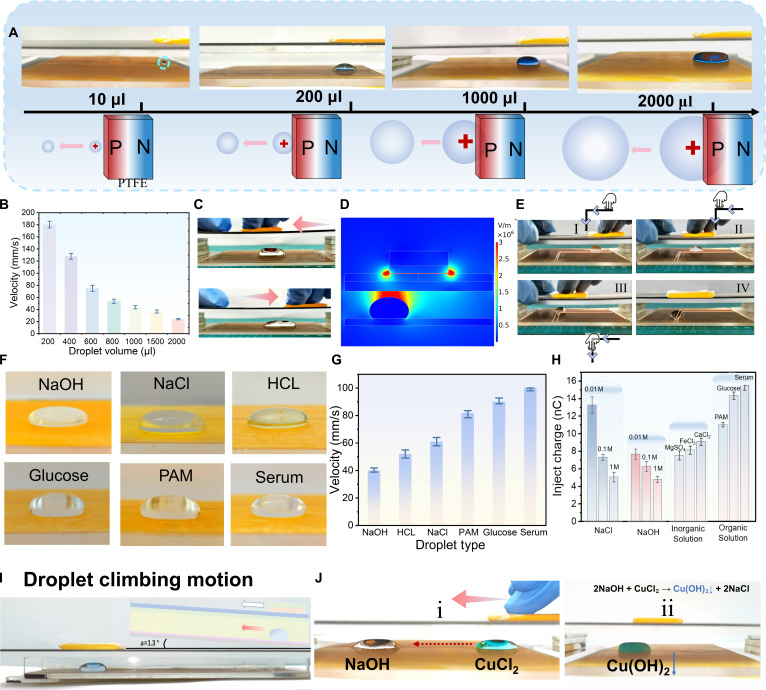
Demonstration for WEDMS’s generality with various liquids, including different droplet volumes and different types of droplets. (A) WEDMS-based actuation of droplets with volumes ranging from microliters to milliliters. (B) Moving velocities of droplets with different volumes. (C) Schematic illustration of charge shuttling for droplet actuation under a bipolar electric field. (D) COMSOL simulation of the electrostatic field in DRP-TENG. (E) Lateral and longitudinal manipulation of droplets using WEDMS. (F) Optical images of different types of droplet driving (600 μl). I-IV denote the sequential steps involved in the sliding process. (G) Moving velocities of droplets with different types. (H) Magnitude of injected charges in different types of droplets on hydrophobic layer. (I) Optical images show the well-controlled transport of water droplets on a sloped surface with an angle of ∼1.3°. (J) WEDMS demonstration of droplets undergoing a merging chemical reaction. Labels (i) and (ii) represent the sequential steps involved in the sliding process.

WEDMS demonstrates universal applicability in driving various droplet volumes and types compared to conventional droplet manipulation methods. As shown in Fig. 3A, under WEDMS technology, droplet volumes range from 10 to 2,000 μl, including deionized water and blue ink, which are successfully driven in real time without cables. Droplets can start and stop immediately with the slider, and is capable of high speeds, unrestricted transport distance, flexible steering, and precise positioning. As the droplet volume increases, the droplet movement speed gradually decreases (Fig. 3B). The velocity of a 200-μl droplet can reach up to 180 mm/s, and is further displayed in Movie S6, exceeding values reported in previous studies. Although larger droplets (2,000 μl) (as shown in Movie S7) can be injected more charge (Fig. S15), their increased mass leads to higher resistance, consequently slowing the droplet movement speed. Under the influence of the electric field, as the droplet volume increases, both the voltage and current show a gradually decreasing trend (Fig. S15). It takes a charge injection time of 1 to 2 s during the early stage of droplet driving, also referred to as the droplet start-up time, and the PTFE slider can also move approximately 2 cm on the PU layer to reach the droplet site, at which point droplet driving begins. As shown in Fig. 3C, when the distance gap exceeds 12 mm, the 1,200-μl droplet will not move with the slider in real time, and the droplet will remain stationary prior to movement. When WEDMS is activated to the left or right, even if the droplet does not depart from its initial position, it will exhibit a tendency to extend in the corresponding direction, visually demonstrating the phenomenon of charge shuttling on droplet actuation in a bipolar electric field. The entire process is comprehensively documented in Movie S8. COMSOL software is employed to simulate the static electric field distribution of DRP-TENG and the droplet assembly (Fig. 3D and Note S5), corresponding to the bottom discharge image captured in Fig. 1E. The simulation confirmed that the strong corona discharge effectively injected charge into the droplet, enabling its controlled actuation. As a result, the droplet can move in any direction under the guidance of the slider. DRP-TENG is able to allow the PTFE slider to drive the droplets in both horizontal and vertical dimensions (Fig. 3E and Fig. S16), enabling the transition of droplet motion from 1 to 2 (Fig. S15) and even 3 dimensions (in oil) under the guidance of the slider. The simplicity and flexibility of the WEDMS system enable real-time, complex droplet steering, a capability difficult to achieve with conventional droplet manipulation system.

Furthermore, the WEDMS system demonstrates versatility in manipulating various types of droplets (Table S1), including inorganic solutions (NaOH, NaCl, HCl), non-surface-active water-soluble organic solution (glucose, polyacrylamide), and serum solutions (Fig. 3F), Such solutions exhibit high surface tension, strong polarity, nonsurface activity, and large contact angle, resulting in poor compatibility with hydrophobic surfaces. They can maintain a stable spherical morphology on hydrophobic surfaces, and precise manipulation of these droplets can be achieved via charge injection and electrostatic force. In addition, the driving speeds for different droplet types are evaluated, revealing that, for the same volume, serum droplets exhibited the highest speed, with a 600-μl droplet reaching 100 mm/s. According to the data, the increased positive charge accumulation observed in serum during the WEDMS sliding process is attributed to electrostatic adsorption onto proteins, lipids, lipoproteins, and other negatively charged biomolecules present in the serum [38,39]. Overall, non-surface-active water-soluble organic solution demonstrated faster motion than inorganic ones (Fig. 3G). This phenomenon is attributed to the fact that, in electrolyte solutions, conductivity depends on ion concentration. Considering the influence of conductivity on charge injection during corona discharge, electrolyte solutions with concentrations of 0.01, 0.1, and 1 M are prepared to obtain varying conductivities, and the corresponding charge injected into droplets under WEDMS operation was measured (Fig. 3H). For example, electrolyte solutions containing Na^+^, OH^−^, and acidic ions can induce charge shielding effects, thereby reducing the surface charge density of droplets [40]. As a result, the electrical output decreases, and the electrostatic force weakens, which ultimately slows down the droplet’s movement speed compared to deionized water. The WEDMS method enables efficient, versatile manipulation of diverse droplets, demonstrating broad applicability in biomedical research, environmental monitoring, and catalytic systems.

### Versatile applications of WEDMS for droplet transport and multiphase reactions

**Fig. 4.**
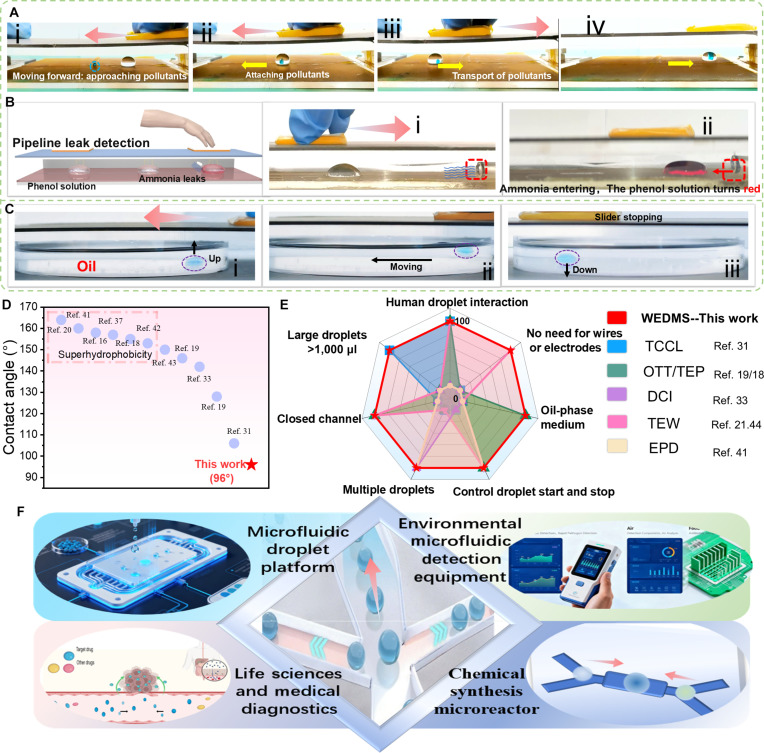
Application demonstrations of WEDMS for driving droplets. (A) Schematic diagram of WEDMS actuating liquid–solid system. (B) Schematic diagram of ammonia detection. (C) WEDMS manipulates droplets in the oil base. (D) Comparison of contact angles with previous works. (E) Comparison between WEDMS and previously reported droplet manipulation performance from 7 perspectives. The evaluations are conducted based on the detailed information in Table S2. WEDMS’s wide operability and universality are demonstrated. (F) Development prospects and outlook of wireless microfluidic systems. Labels (i) - (iv) in the figure represent the sequential steps involved in the sliding process.

The coulomb force generated between TENG and the droplet not only drives droplets on smooth, horizontal surfaces but also enables WEDMS to propel them along inclined channels with a slight tilt of 1.3° (Fig. 3I), achieving a transport distance of 13 cm within just 1 s. Beyond droplet climbing, WEDMS also serves as a platform for chemical reactions, precisely manipulating CuCl₂ droplets for coalescence (Fig. 3J and Movie S9) and facilitating additional reactions such as color changes (Fig. S17), precipitation, and charge-mediated processes. Moreover, WEDMS can synchronously actuate liquid–solid systems, enabling coordinated movement of droplets and solid objects. In Fig. 4A and Movie S10, (i) under WEDMS drive, the droplet moves leftward along with the slider; (ii) upon contact, the droplet merges with the solid object and both continue to move to the left; and (iii) when the slider reverses direction, the liquid–solid composite likewise moves rightward, following the slider’s motion. WEDMS technology is also capable of precisely manipulating droplets within enclosed channels. In a sealed environment, a phenolphthalein droplet can be accurately guided, while ammonia gas is gradually introduced from one end (Fig. 4B and Movie S11). As the droplet approaches the gas inlet, its color progressively changes from colorless to red (Fig. S18). This approach demonstrates significant potential for environmental monitoring, particularly in identifying localized ammonia leaks in confined pipeline infrastructures.

The WEDMS droplet-driving method demonstrates broad universality, enabling not only droplet actuation in air but also effective manipulation within an oil matrix, thereby achieving 3-dimensional droplet motion. In this setup, the TENG structure remains unchanged, with its lower section modified into an insulated, enclosed chamber filled with dimethyl silicone oil as the droplet transport medium. The experimental setup for droplet manipulation in an oil chamber is depicted in Fig. S19. The droplet movement in the oil base primarily involves 3 stages (Fig. 4C and Movie S12): (a) The PTFE slider moves approximately 2 cm on the PU (droplet start-up), reaching the droplet’s vertically above position and injecting positive charge, which generates a strong electrostatic force initiating droplet ascent [Fig. 4C(i)]. (b) When the combined electrostatic and buoyancy forces exceed the gravitational force, the droplet rises to the 3-phase interface, where it partially emerges from the oil, stabilizes, and begins to move horizontally with WEDMS [Fig. 4C(ii)]. (c) The strong electrostatic force between the droplet and WEDMS drives the droplet along the 3-phase interface. Once guided to the target position, the slider stops, the electrostatic force is removed, and the droplet, under gravity, freely descends back into the oil medium [Fig. 4C(iii)], and the force analysis in oil layers is depicted in Fig. S20 (Note S6). The resistance of the oil matrix reduces droplet movement speed compared to air. The oil-phase driving strategy of WEDMS is also applicable to various oil-phase systems, including vegetable oil and mineral oil, with detailed results shown in Fig. S21. However, dimethylsilicone oil, due to its high dielectric constant and low viscosity, exhibits the fastest response rate and best driving performance among the 3 oil-phase media. However, droplet manipulation in oil effectively prevents cross-contamination, suppresses dispersion, and inhibits evaporation. Overall, the proposed WEDMS system exhibits outstanding performance in droplet control, overcoming spatial and environmental limitations while offering simple operation, high adaptability, wireless integration, and excellent human–droplet interaction flexibility.

As shown in Fig. 4D, in most previously reported electrically controlled droplet systems [16,18,20,31,33,37,41–43], the contact angle between droplets and hydrophobic surfaces typically exceeds 100°. In this work, droplets with contact angles as low as 96° and volumes up to 800 μl are stably and continuously driven. Even larger droplets with lower contact angles maintained reliable actuation, demonstrating the excellent adaptability and versatility of the WEDMS system. Moreover, as summarized in Fig. 4E [18,19,21,31,33,41–44], WEDMS technology outperforms conventional droplet manipulation methods in multiple aspects, owing to its unique actuation mechanism and precise control capability. From the technical perspective, the WEDMS system holds significant potential for both technological advancement and practical applications. Its simplicity and modularity, combined with wireless operation and stable actuation of multiple droplet types, make it highly promising for biomedical analysis, drug delivery, contamination-free chemical synthesis, environmental monitoring, water-quality microfluidic chip analysis, and integrated microfluidic systems (Fig. 4F). In the future, to further enhance its applicability across multidisciplinary scenarios, hydrophobic interfaces with improved performance and greater stability could be developed. Such interfaces would increase compatibility with diverse droplet samples, particularly those involving hazardous, toxic, or sensitive reagents, thereby effectively expanding the system’s practical applications and overall utility. This advancement will provide an innovative technological solution for microfluidics and droplet manipulation, thereby promoting the development and application of these related fields.

## Conclusion

In summary, this study presents a dual-region dynamic electric field regulation mechanism at the friction interface, which enables precise dual-region electric field control and corona discharge-induced droplet charge injection solely via a flexible, electrode-free friction interface, without the need for electrode arrays or external circuits. This innovative strategy effectively couples the dynamic electric field structure of the triboelectric layer with droplet manipulation functions, fundamentally eliminating the structural constraints imposed by conventional electrodes and circuits, and significantly enhancing the flexibility and responsiveness of droplet control. Because of the unique structural design and the synergistic effects of dielectric polarization and local corona discharge, DRP-TENG can directly inject charges into target droplets, thereby generating coulombic attraction or repulsion. This system enables continuous, real-time manipulation of droplets ranging from 10 to 2,000 μl, driven by simple manual mechanical actuation, with a maximum speed of 180 mm/s for 200-μl droplets. It efficiently handles high-surface-tension liquids such as acids, bases, salts, and nonsurfactant water-soluble organic solvents. The technology is stable in closed environments, on inclined surfaces, and in oil-phase media. Its simple structure and ease of modular integration offer a promising platform for microfluidics and related applications, advancing both research and practical use in these fields.

## Materials and Methods

### Manufacturing of the WEDMS droplet manipulation system

The WEDMS system is integrated with 2 main structures, with the upper part being the hand control platform integrating DRP-TENG and the lower part being the droplet control platform, leaving a gap of 10 mm between the 2 platforms. DRP-TENG for the manual control platform: The tribo-materials used in DRP-TENG are PTFE and PU, with thicknesses of 0.05 and 1 mm respectively. An acrylic sheet (3 mm in thickness) was cut into rectangular plates of 25 mm × 35 mm and 200 mm × 100 mm by a laser cutter. The droplet manipulation platform consists of quartz glass coated with a superhydrophobic layer (Narroco NC319, purchased from Naroco New Materials Technology Co. Ltd., Changzhou), with the glass measuring 10 mm × 10 mm. Hydrophobic coating is applied on the glass, which is then placed in a drying cabinet for 10 min to form a thin and uniform-shaped hydrophobic layer. In the experiment, deionized water droplets are used, and to facilitate observation, blue ink is added to the manipulated droplets.

### Measurement and characterization

The surface micro-nano morphology of the PU foam, PTFE film, and hydrophobic layer was characterized using a field emission scanning electron microscope (Germany ZEISS Sigma 300). A purple glow discharge between TENG and droplets was captured using a digital camera (Fuji). The contact angle of droplets on the hydrophobic surface and the dynamic behavior of the droplets in air and oil phase were recorded using a smartphone camera (iPhone 16 Pro Max). Electrical signal outputs from DRP-TENG and the charge within the droplets were measured using programmable electrometers (Keithley 6514). The distribution of the electric field was simulated using COMSOL multiphysics software.

## Data Availability

All data are available in the main text and the Supplementary Materials. Additional data are available from C.S. (chuncaishan@kust.edu.cn or panbocai@aliyun.com) upon reasonable request.

## Supplementary Materials

Supplementary 1 Figs. S1 to S24 Notes S1 to S6 Tables S1 and S2 Movies S1 to S12
