# Supplementary material for: Non-Electrode Droplet Manipulation via Triboelectrification Near-Field Energy Transmission
Source: Research (Wash D C). 2026 May 12;9:1277. doi: 10.34133/research.1277 (PMC13161535; doi:10.34133/research.1277)
Supplement: Supplementary 1 — Figs. S1 to S24 Notes S1 to S6 Tables S1 and S2 Movies S1 to S12 [file research.1277.f1.zip › Supplementary information.docx]

**Supplementary Information**

Non-Electrode Droplet Manipulation via Triboelectrification Near-Field Energy Transmission

Jun Han^1^, Ruxue Huang^1^, Chuncai Shan1*, Kaixian Li^2^; Huiyuan Wu^2^; Peng Zhang^1^, Wei Long^3^, Haifeng Qian^3^ ,Sichen Lu^3^, Bo Pan^1*^, Chenguo Hu^2*^

Chuncai Shan, Bo Pan

Yunnan Provincial Key Laboratory of Soil Carbon Sequestration and Pollution Control Faculty of Environmental Science&Engineering, Kunming University of Science&Technology, Kunming 650500, P. R. China
E-mail: [chuncaishan@kust.edu.cn](mailto:chuncaishan@kust.edu.cn), [panbocai@aliyun.com](mailto:panbocai@aliyun.com)

Chenguo Hu

School of Physics, Chongqing University, Chongqing 400044, P. R. China

E-mail: [hucg@cqu.edu.cn](mailto:hucg@cqu.edu.cn)

**Contents**

**Figure S1.** Schematic illustration of all kinds of Droplet manipulation methods.

**Figure S2.** Diagram of the experimental device for WEDMS manipulation of droplets.

**Figure S3.** SEM images of tribo-materials PU and PTFE.

**Figure S4.** Comparison of contact angles at different interfaces（800 μL droplet）.
**Figure S5.** The output charge of the DRP-TENG triboelectric interface corona discharge when varying atmospheric relative humidity.

**Figure S6.** The long-term operational stability of the system.

**Figure S7.** Diagram of the opposite output performance structure on both sides of the front and rear of the detection slider (a-b) and Equivalent physical pattern(c).

**Figure S8.** The slider drives both droplets at the same time and COMSOL software simulation of the electrostatic field distribution and droplet assembly of two droplets.

**Figure S9.** The charge retention in the droplets.

**Figure S10.** Change in droplet charge when the slider repeatedly passes over the same droplet.

**Figure S11.** COMSOL software is employed to simulate the static electric field distribution of the DRP-TENG and the droplet assembly in the different distance h.

**Figure S12.** Electrical signal output at different distances. (a) Voltage signals in droplets at different distances. (b)Current signals in droplets at different distances.

**Figure S13.** The voltage and current output signals of the DRP-TENG.

**Figure S14.** (a)Four different substrate materials (b)The voltage values(c) The output charge of TENG with different substrate materials.

**Figure S15.** (a) Relationship between droplet volume and injected charge. (b) Correlation between droplet volume and electrical output performance (voltage/current).

**Figure S16.** (a) The motion trajectories of the slider and the droplet. (b)The operating mode of DRP-TENG is upgraded from one-dimensional linear motion to two-dimensional planar motion.

**Figure S17.** (a) Photograph of Cu (OH)_2_ precipitation. (b) The color reaction between NaOH and phenolphthalein droplets,

**Figure S18.** Diagram of the experimental device for ammonia monitoring of droplets.

**Figure S19.** Experimental setup of the oil chamber.

**Figure S20.** Schematic representation of WEDMS manipulation of droplets within oil layers and Force analysis related to WEDMS manipulation of droplets in oil layers.

**Figure S21.** Motion states of droplets in four different oils.

**Figure S22.** Hard and soft contact of friction materials.

**Figure S23.** Sources of charge in droplet.

**Figure S24.** Simulated electric field intensity inside the materials during the WEDMS process.

**Note S1.** The soft contact increases the effective area of the interface, which significantly improves the output performance of the DRP-TENG.

**Note S2.** In the WEDMS system, the injected charge plays a dominant role in droplet manipulation.

**Note S3.** The DRP-TENG slider presents a dual-region opposite-polarization to drive two droplets at the same time.

**Note S4.** The breakdown voltage of the WEDMS system is calculated according to Paschen's law.

**Note S5.** The distribution of electric field intensity across different materials during discharges facilitated by dielectric polarization.

**Note S6.** Force analysis related to WEDMS manipulation of droplets in oil layers.

**Table S1.** The surface tension and the sphericity characteristics exhibited by the droplet samples that can be driven by the WEDMS system.

**Table S2.** Performance comparison of electronically controlled droplets

**Other Supplementary Material for this manuscript includes the following:**

Supplemental Movies S1 to S12


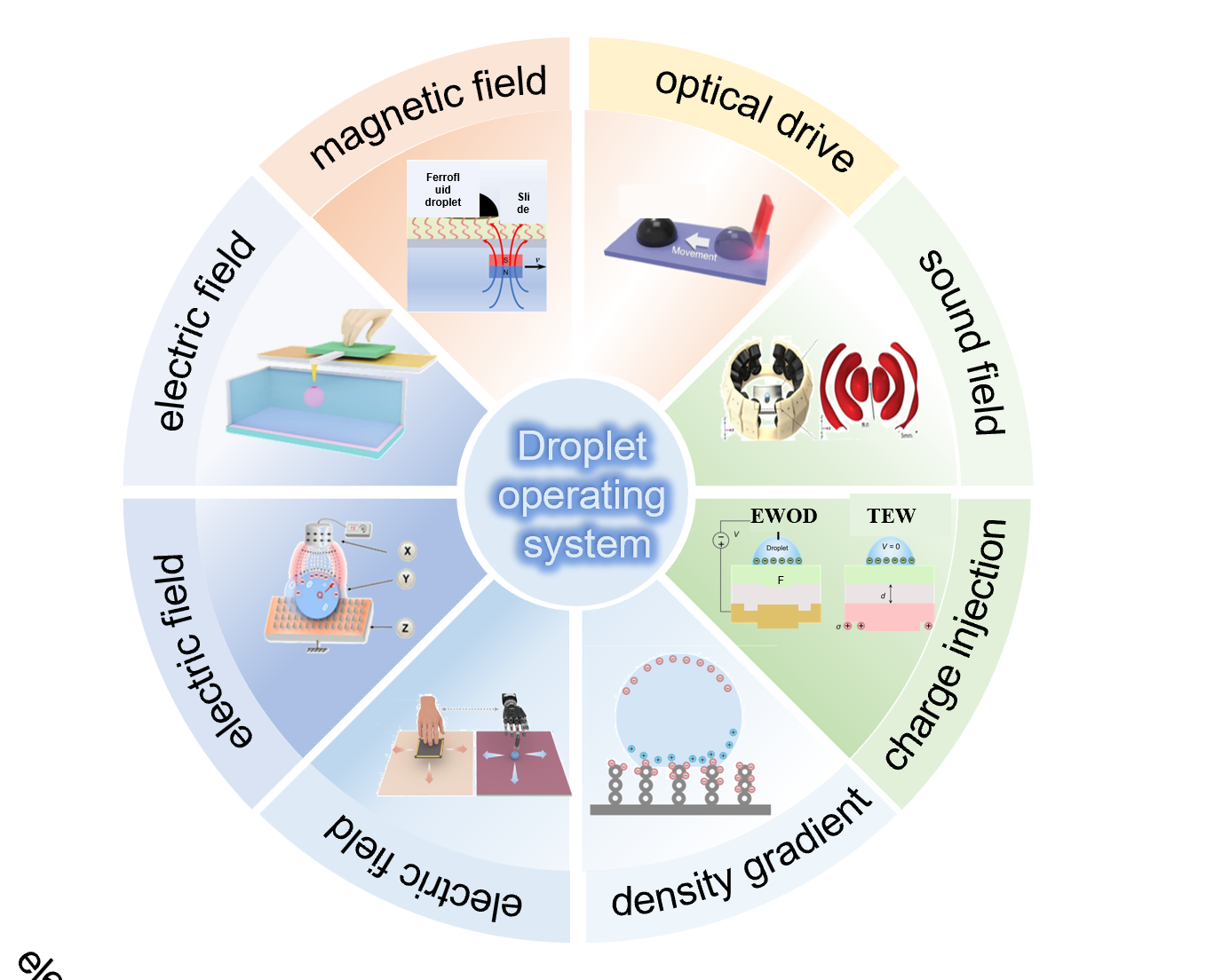


**Figure S1. Schematic illustration of all kinds of Droplet manipulation methods.**


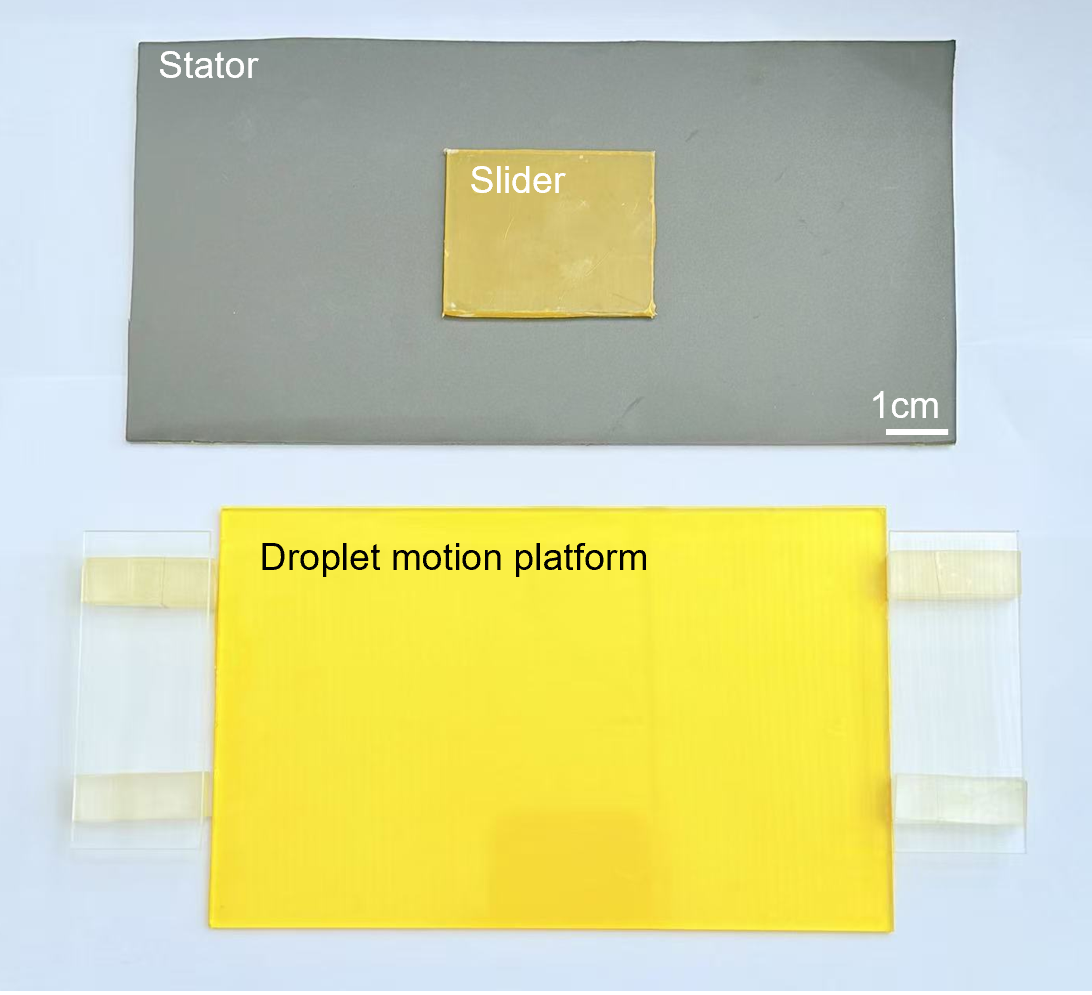

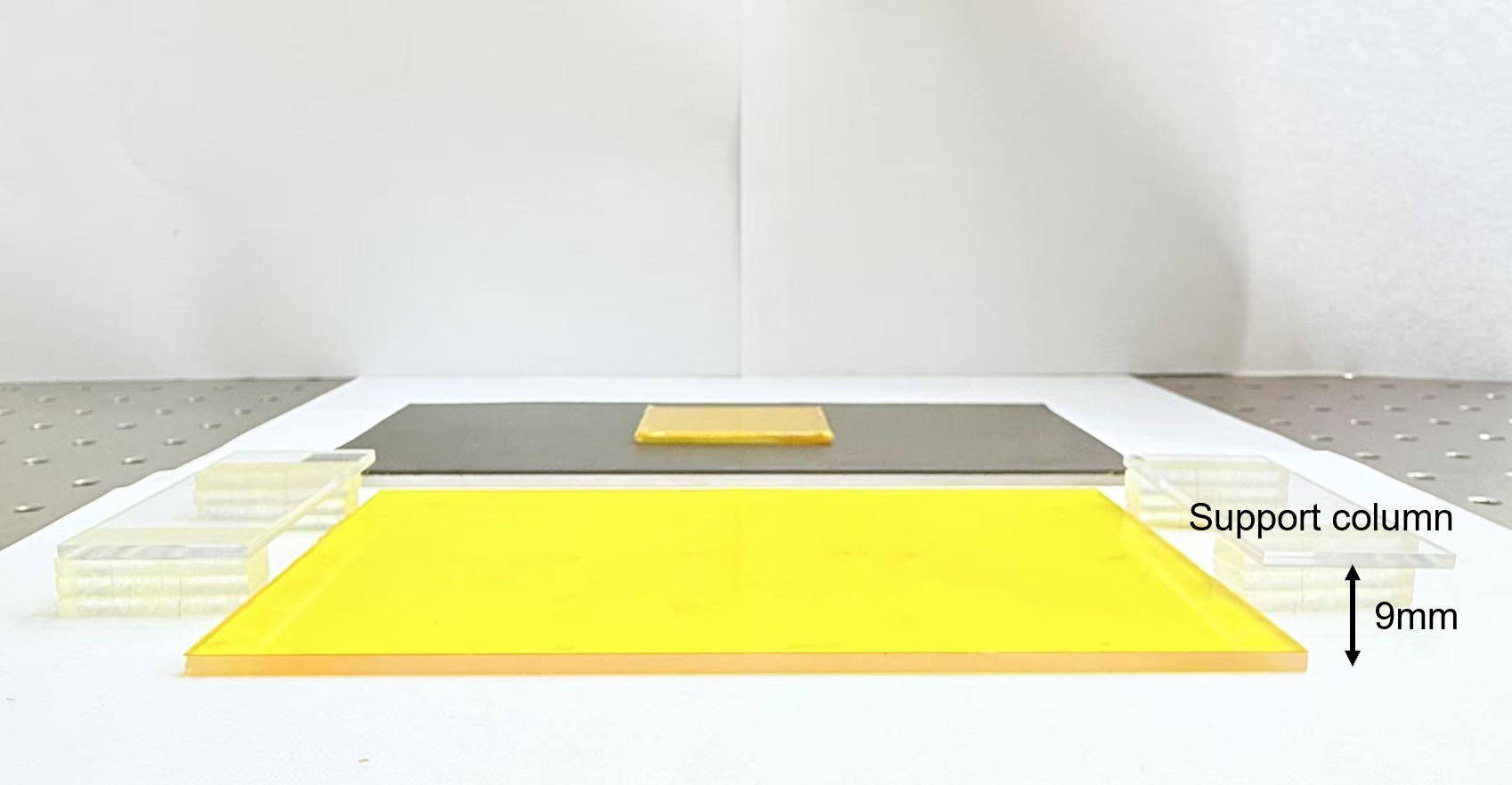


**Figure S2. Diagram of the experimental device for WEDMS manipulation of droplets.** The WEDMS device is composed of a DRP-TENG control platform and a droplet motion platform, among which the DRP-TENG platform is located above the droplet motion platform，it is 9mm from the droplet operating platform.


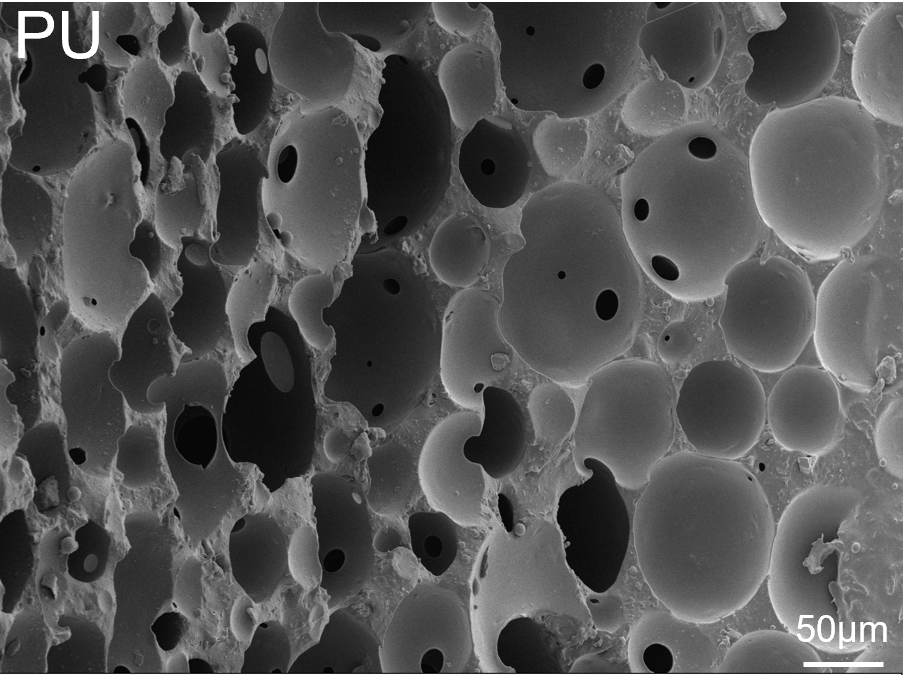

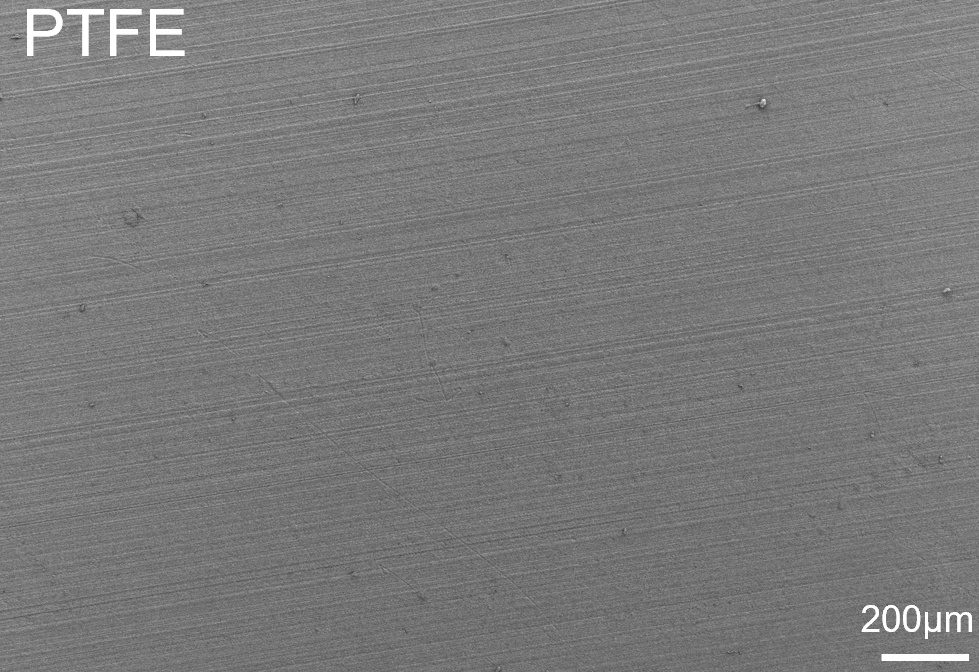


**Figure S3. SEM images of tribo-materials PU and PTFE.** The thickness of the PU foam is 1 mm, and the thickness of the PTFE film is 50μm, all of them are attached to acrylic boards of the same size.


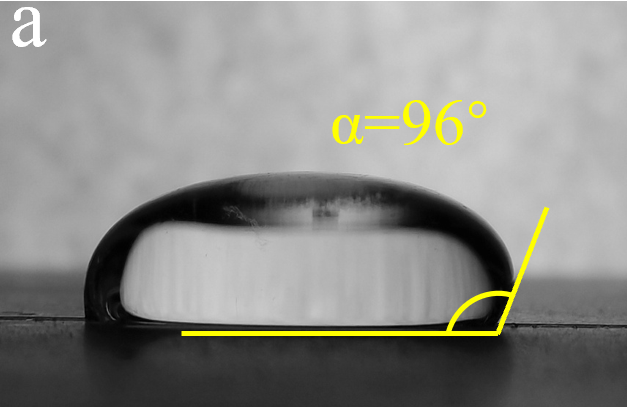
 **
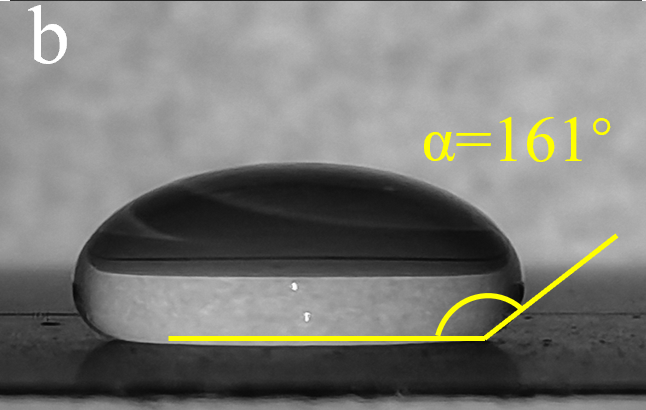
**

**Figure S4.** **Comparison of contact angles at different interfaces（800μL droplet）.** (a)Contact angle on the hydrophobic layer with hydrophilic lines. (b) Contact angle on the hydrophobic layer.


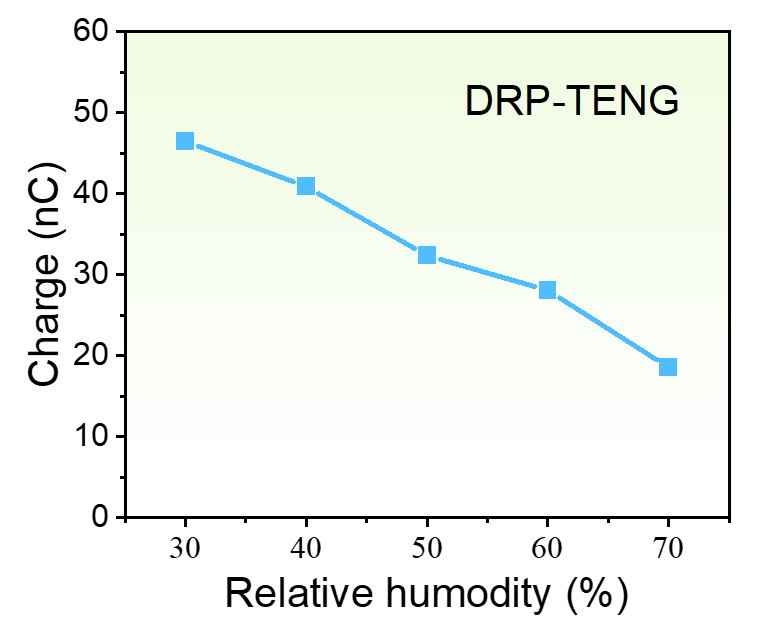


**Figure S5.** The output charge of the DRP-TENG triboelectric interface corona discharge when varying atmospheric relative humidity. The output charge (per cycle) of the DRP-TENG triboelectric interface corona discharge decreases with increasing relative humidity. As humidity rises, water molecules adsorb onto the acrylic surface, forming a conductive water film that increases surface conductivity and accelerates the leakage of triboelectric charges. This reduces the surface charge density and the electrostatic driving force, thereby suppressing the droplet actuation at a humidity level of 70%.


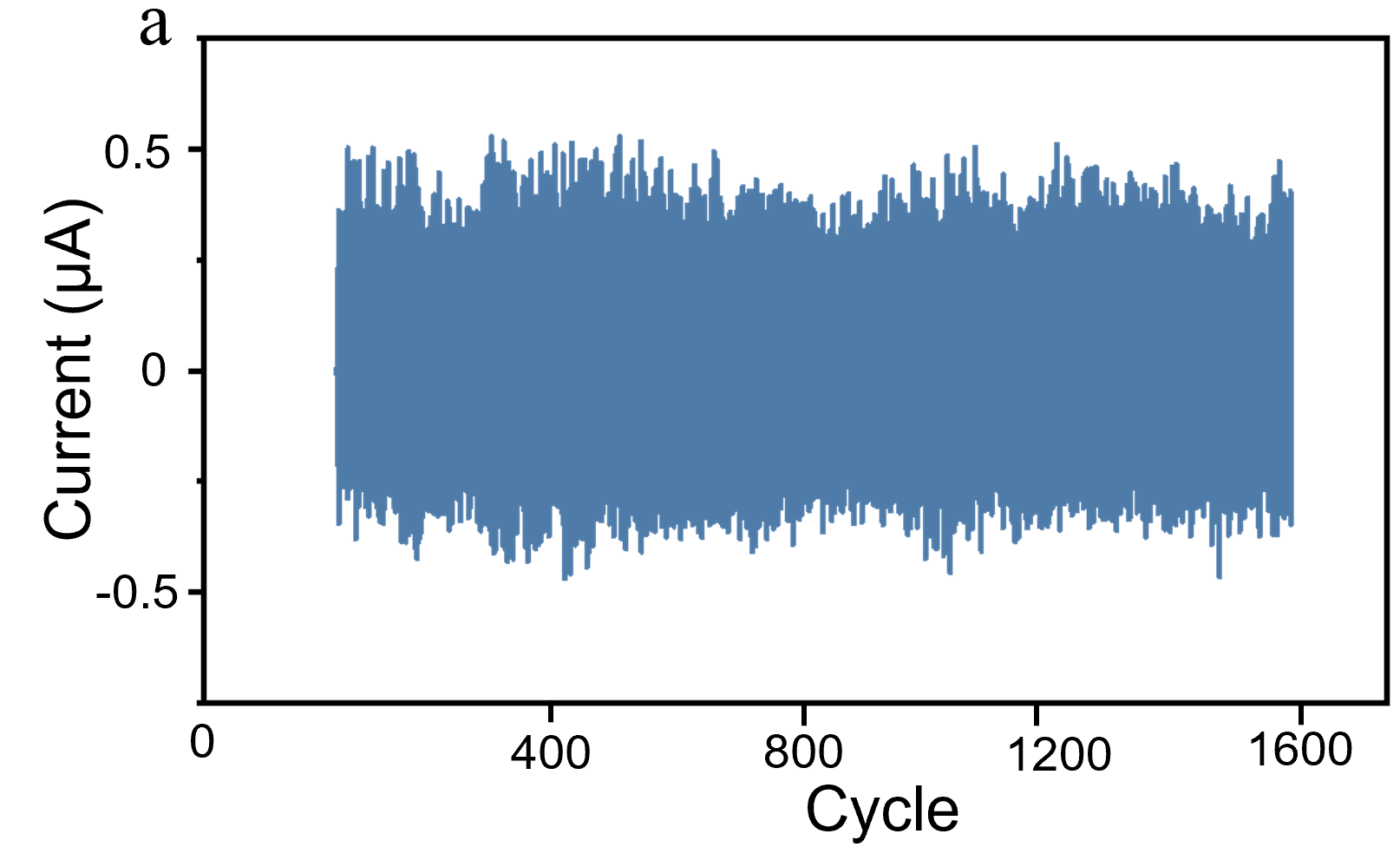


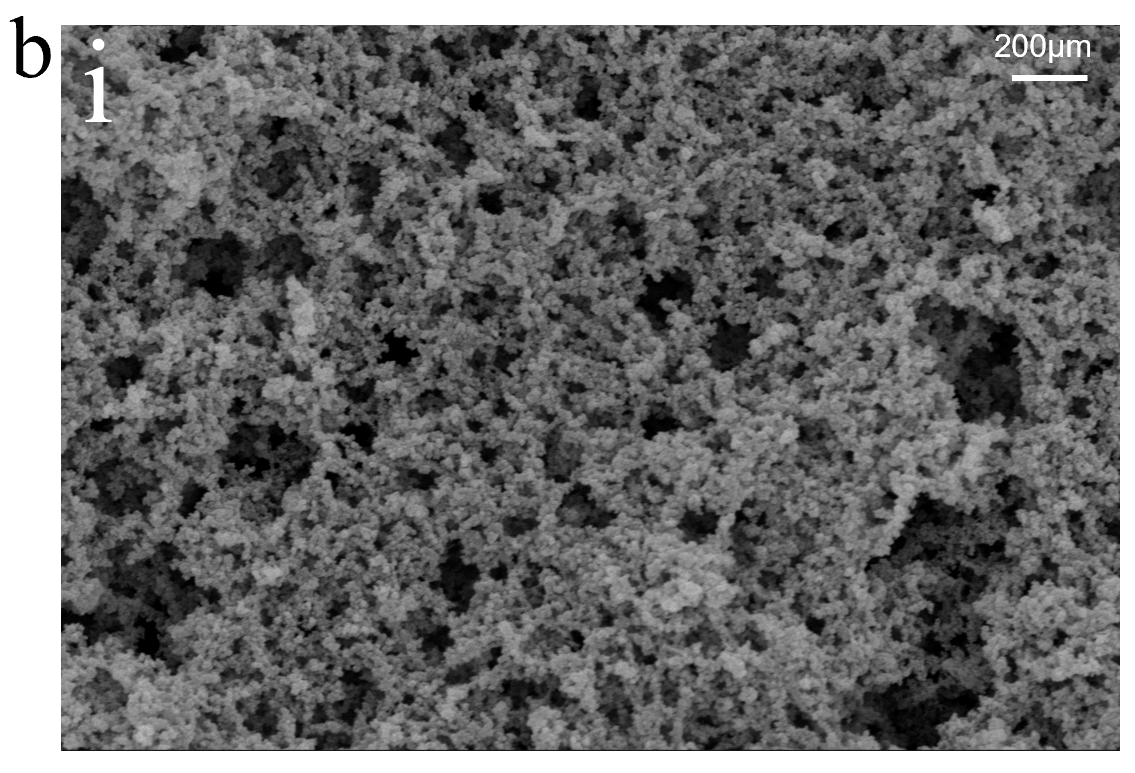

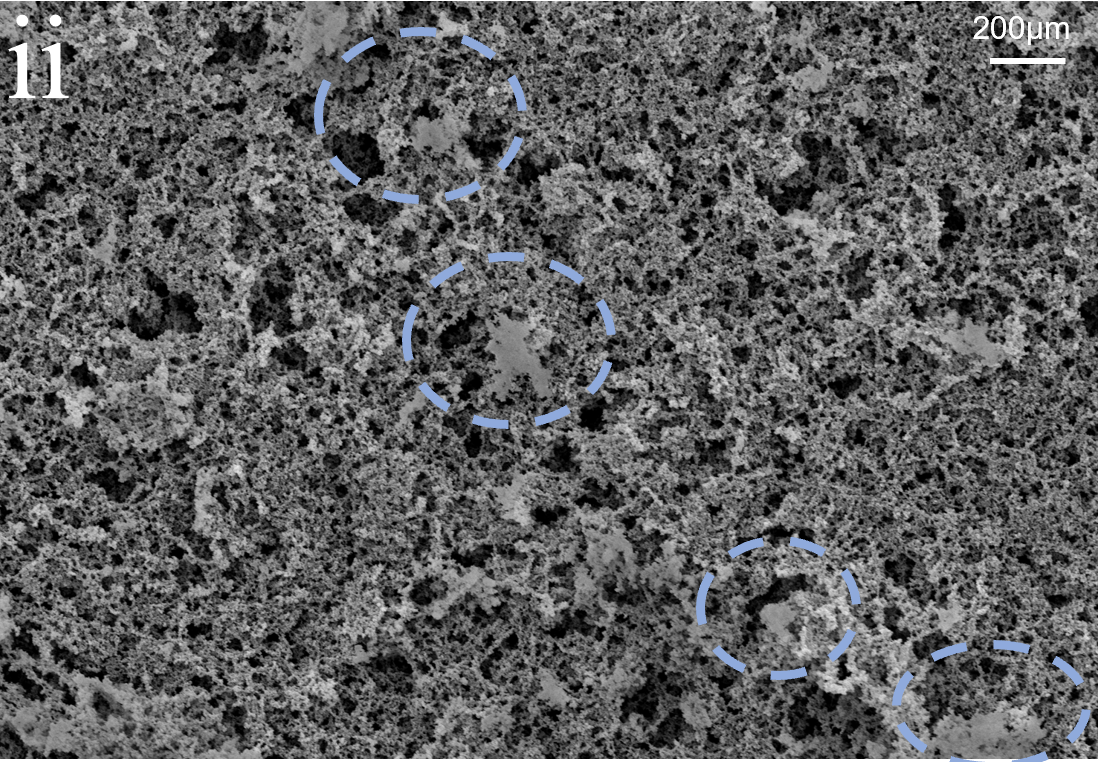


**Figure S6.** **The long-term operational stability of the system.** (a) Operational stability of the DRP-TENG after 1600 reciprocating cycles. After 1600 reciprocating cycles, the output current of the DRP-TENG remains stable. (b) SEM images of the NC319 hydrophobic coating before and after of cyclic use:(i) Before use; (ii) After the droplet repeatedly contacts and rolls along an 8 cm path for 700–800 cycles on the hydrophobic surface. Localized wear and delamination indicate hydrophobic layer detachment, reducing hydrophobicity, triggering local hydrophilic behavior, and impairing droplet operation. Future work should focus on developing more durable hydrophobic layers for high-cycle applications.


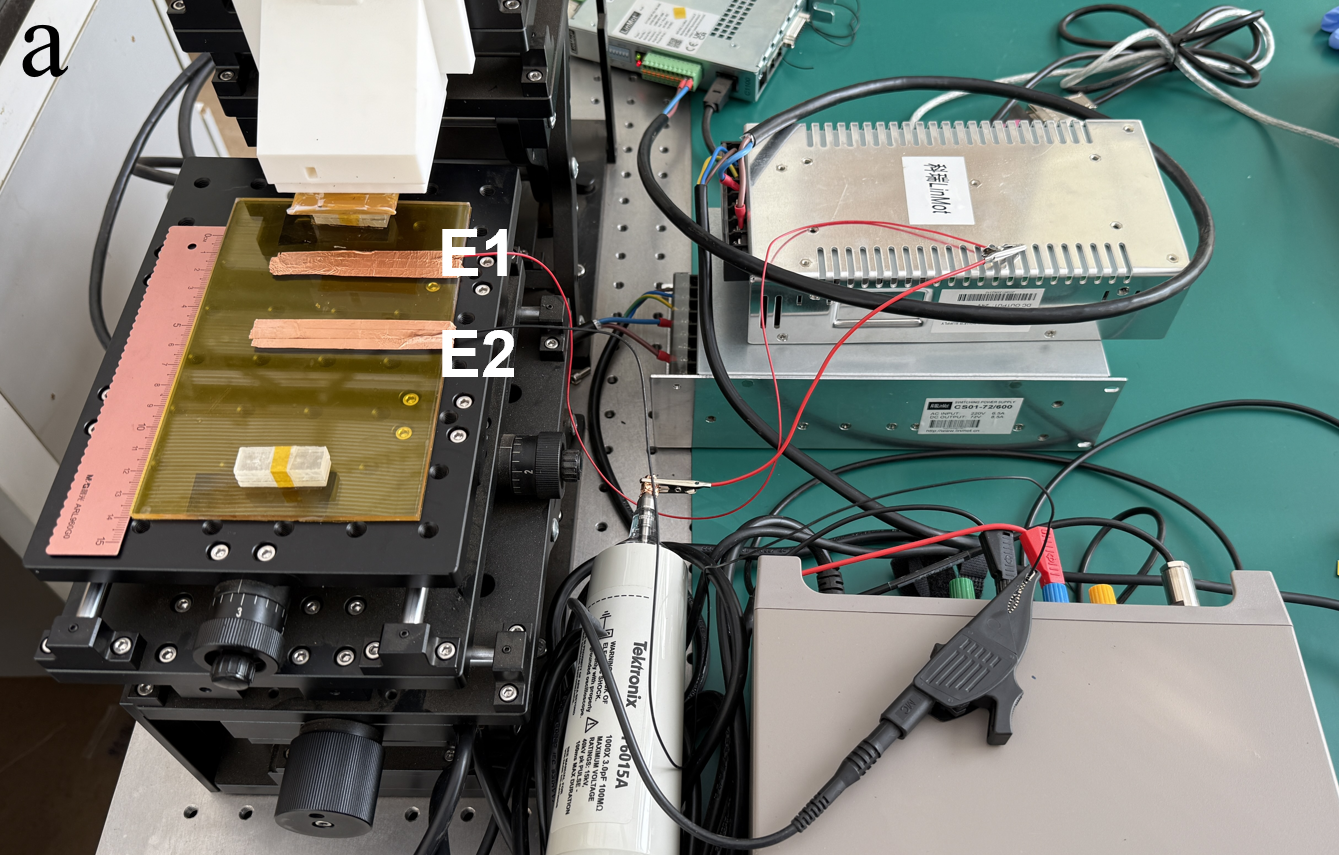

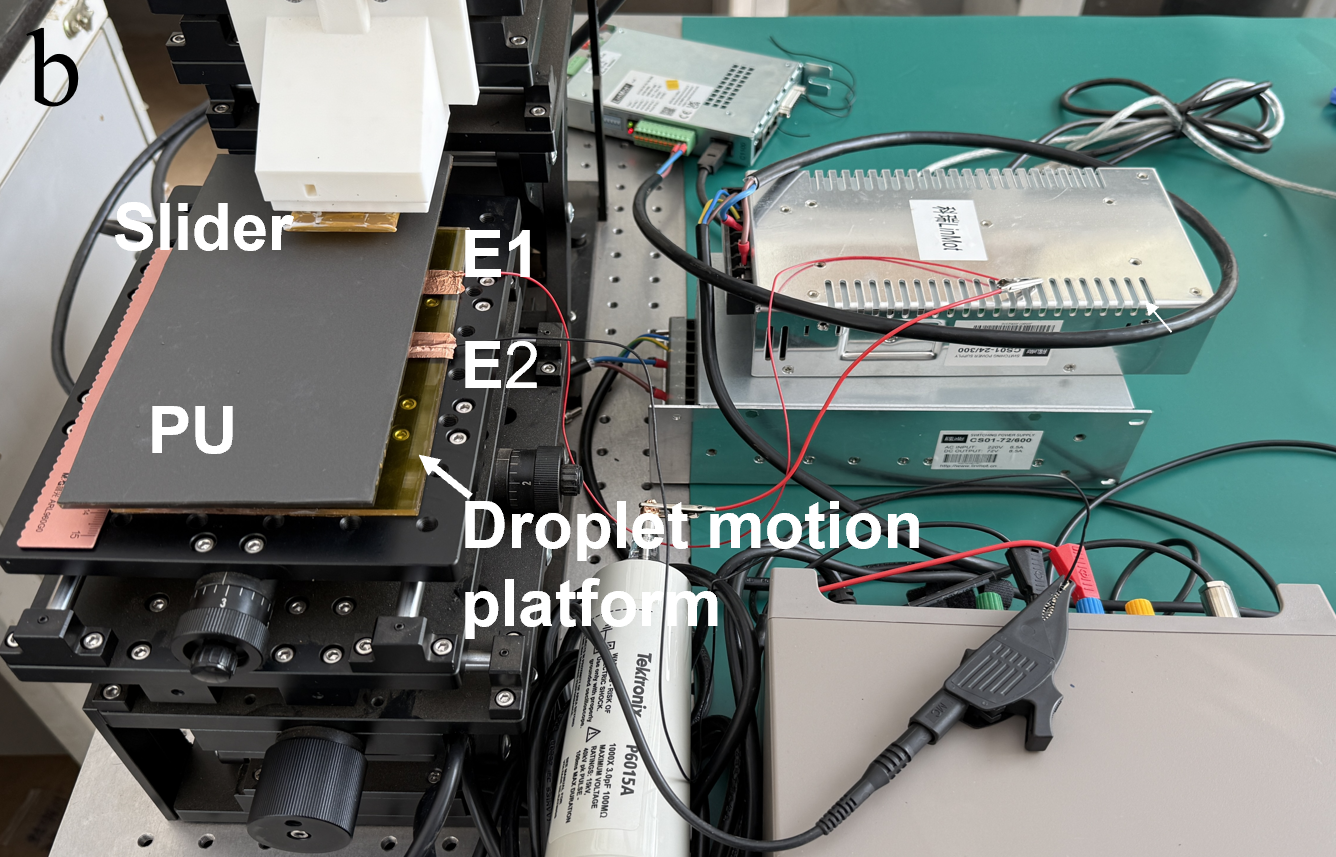


**
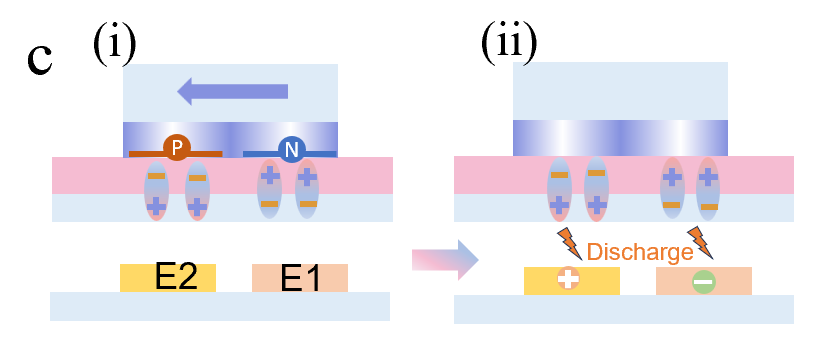
**

**Figure S7.** **Diagram of the opposite output performance structure on both sides of the front and rear of the detection slider (a-b) and** **Equivalent physical pattern(c).** The 6514 electrometer is connected to electrodes E1 and E2, which are positioned on the droplet manipulation platform. When the slider slides in one direction from right to left, the E1 and E2 electrodes at the bottom present the opposite signal phase, verified the previous hypothesis. The independent static electrodes E1 and E2 are attached to the droplet driving layer, and the distance between E1 and E2 is equal to the distance between the front and rear ends of the slider.

**
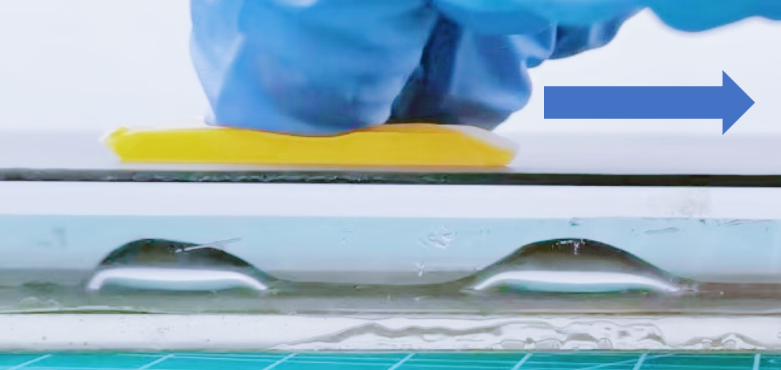

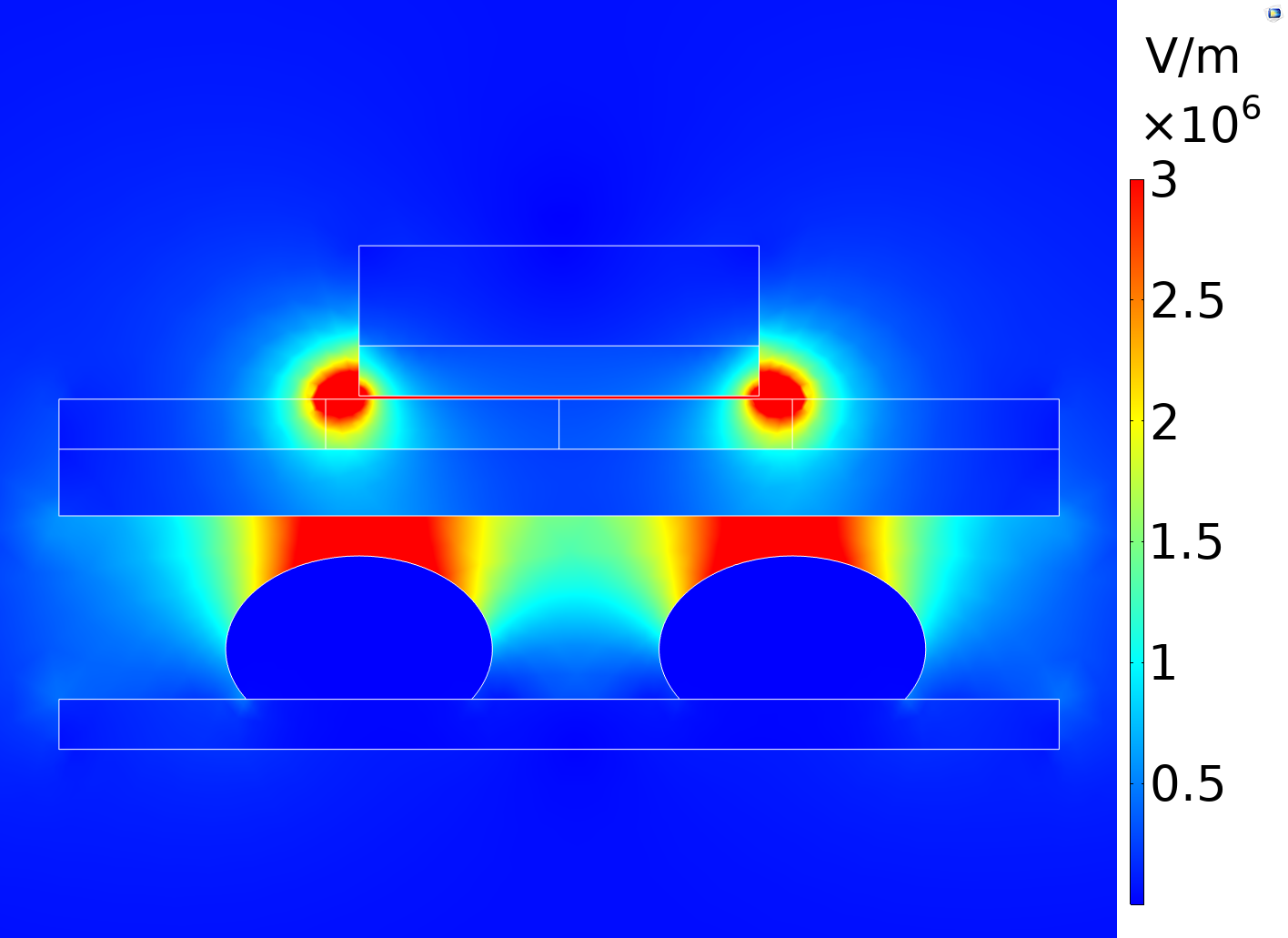
**

**Figure S8. The slider drives both droplets at the same time and COMSOL software simulation of the electrostatic field distribution and droplet assembly of two droplets.**


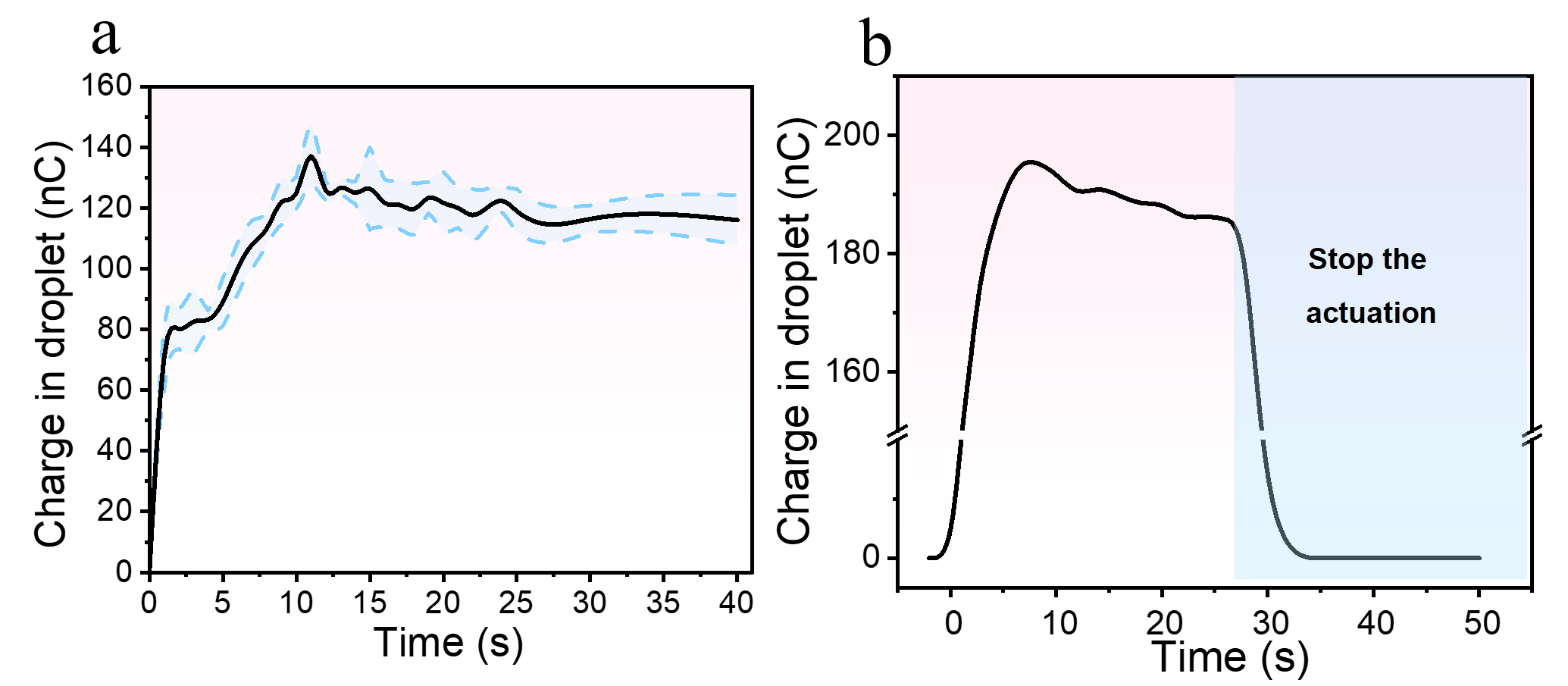


**Figure S9.** (a) The amount of charge injected into the droplet increases initially, then slightly decreases, and finally stabilizes with increasing sliding time (1000 μL). (b) Variation of the droplet charge over time after the driving motion is stopped (1500 μL). When the driving force is suddenly stopped and the external electric field disappears, the electric field strength rapidly drops below the threshold required to maintain gas ionization. As a result, the corona discharge will quickly cease and cannot sustain itself, leading to the sudden dissipation of the charge within the droplet.

**
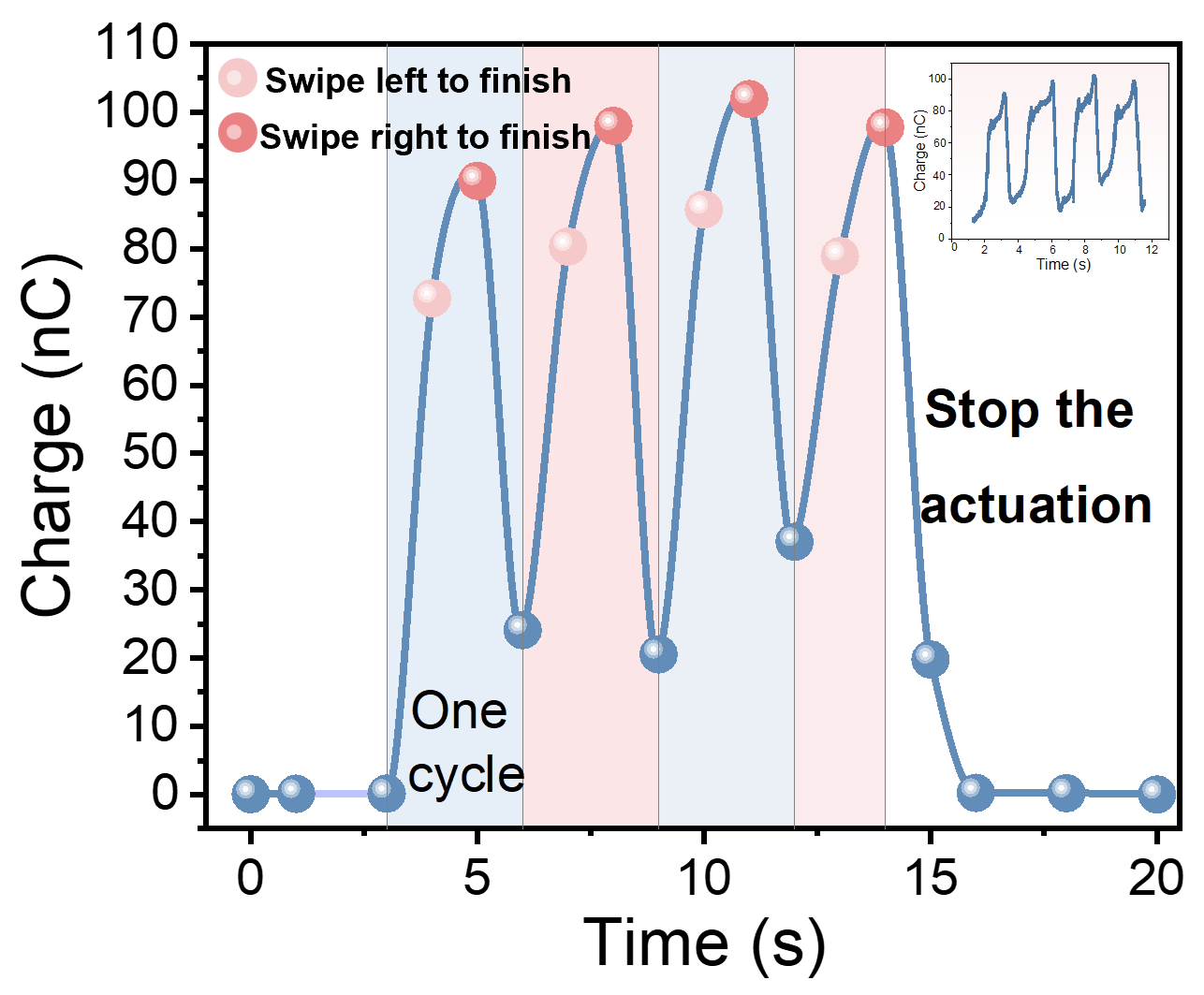
**

**Figure. S10. Change in droplet charge when the slider repeatedly passes over the same droplet. One cycle: The left and right ends of the slider sequentially pass the same droplet and complete one reciprocating motion respectively.** When the gap exceeds 12, the charge carried by the droplet accumulates with each reciprocating motion of the polytetrafluoroethylene (PTFE) slider (one cycle). As the negatively charged end of the slider passes near the droplet, the potential of the positively charged droplet is higher than the surrounding negative potential. As a result, the slider does not inject negative charge into the droplet but instead shows an attraction to the positively charged droplet.

**
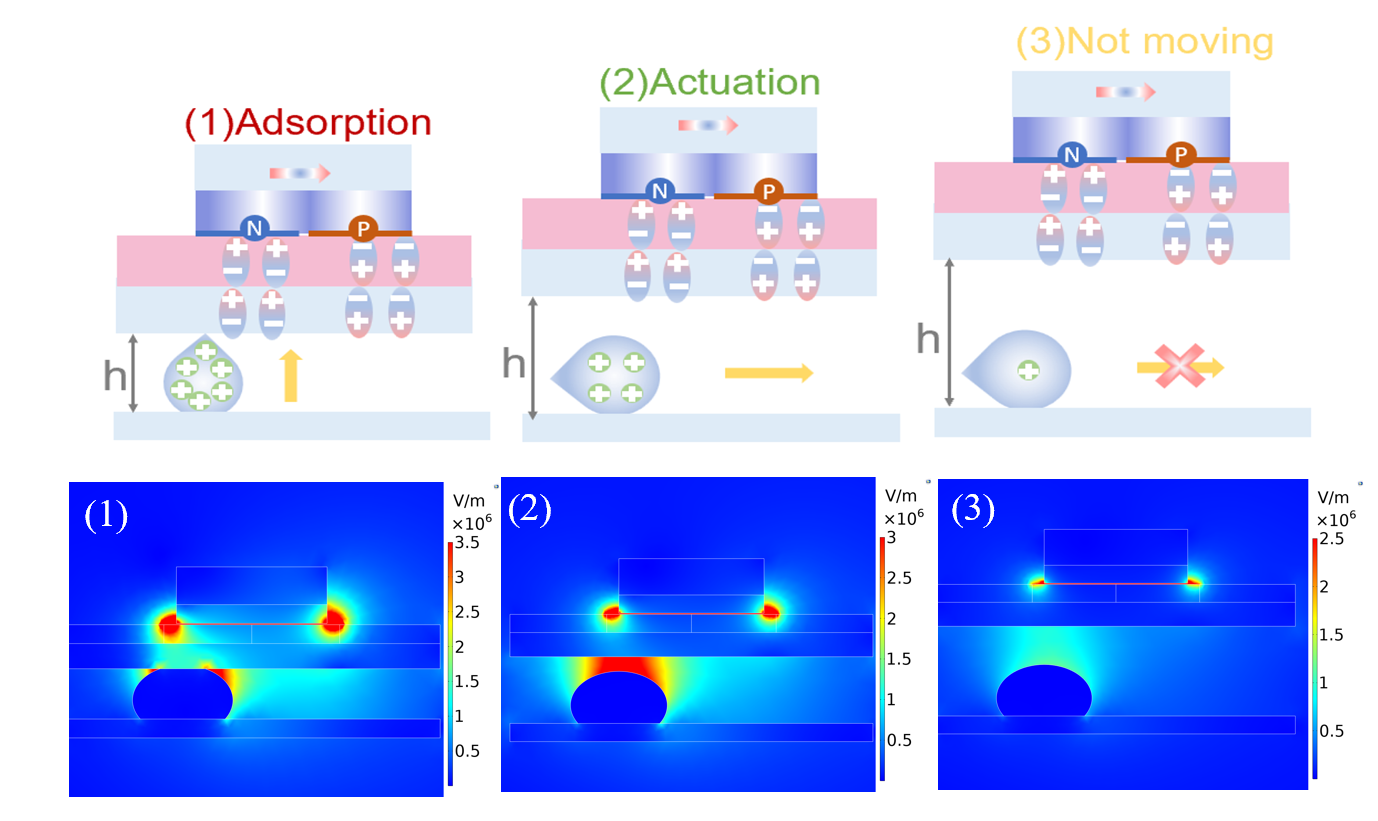
**

**Figure S11.** **COMSOL software is employed to simulate the static electric field distribution of** **the DRP-TENG and the droplet assembly in** **the different distance h.**（1）The small spacing generates a large electric field and a large Coulomb force, which makes the droplets adsorbed on the substrate and cannot be driven.（2）Under the appropriate working spacing, the electric field force is sufficient to overcome frictional resistance and successfully drive the droplet.（3）The spacing is too large, causing air discharge, causing energy loss, and the electric field strength is small, making it impossible to successfully drive the droplet.

①In the gap range of 1–6 mm, due to the small gap, the corona discharge intensity is relatively strong, causing excessive charge capture by the droplet, which easily gets adsorbed onto the friction layer substrate, leading to driving failure. ②In the gap range of 6–12 mm, the charge decay rate is relatively mild, allowing the droplet to be stably and effectively controlled. ③When the effective gap exceeds 12 mm, the air breakdown process becomes unstable as the distance increases, significantly reducing the charge carried by the droplet, ultimately resulting in droplet driving failure.

**
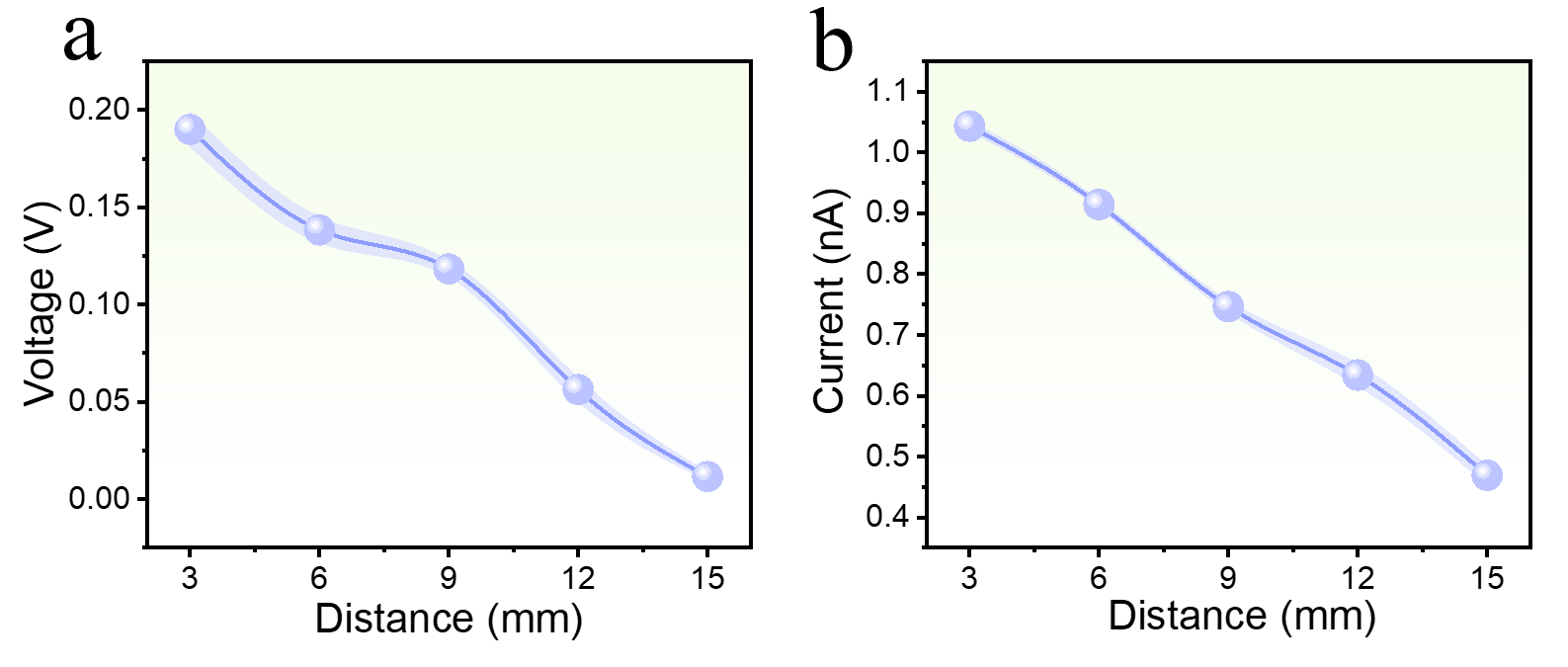
**

**Figure S12.** **Electrical signal output at different distances. (a)** **Voltage signals in droplets at different distances. (b)Current signals in droplets at different distances.**

As the distance increases, the voltage and charge present a gradual decrease trend.

**
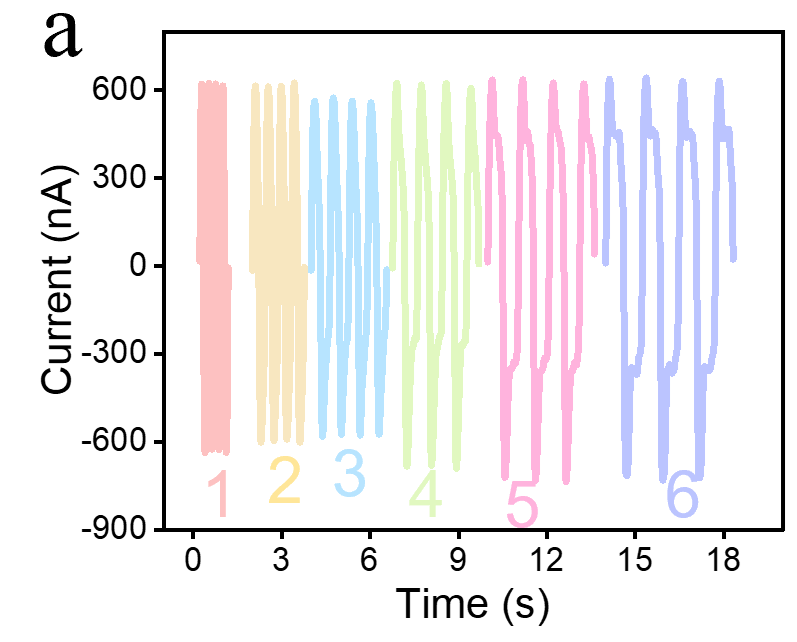

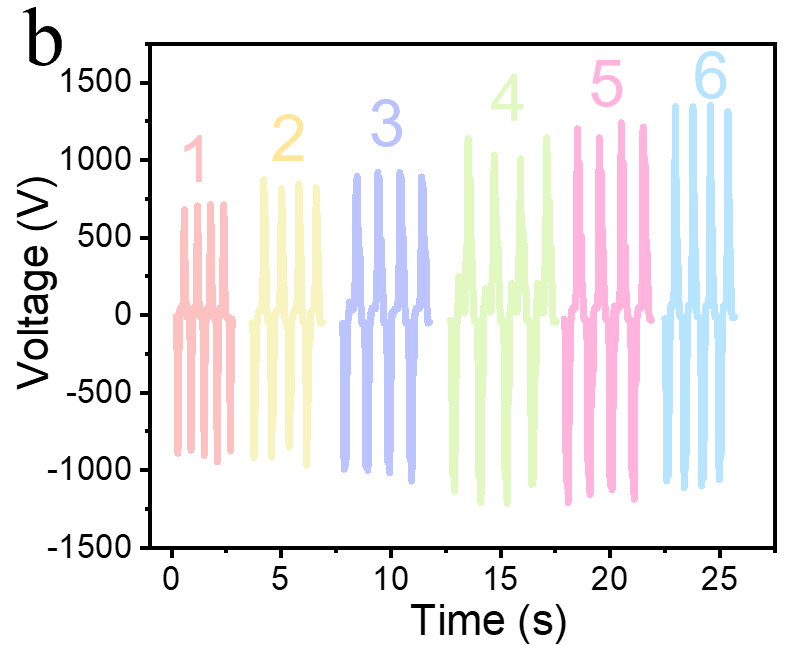
**

**Figure S13.** **(a)The output current of DRP-TENG at different sliding distances(h=9mm).** Since DRP-TENG has AC characteristics, with the increase in sliding distance, the sliding time is prolonged and the pulse width becomes wider. (b) The output voltage of DRP-TENG at different sliding distances(h=9mm). The frictional charge generated by the triboelectric material of the DRP-TENG during the sliding process accumulates progressively with the increase in sliding distance. As a result, a longer sliding distance leads to more accumulated charge at the interface, which increases the interface voltage.

**
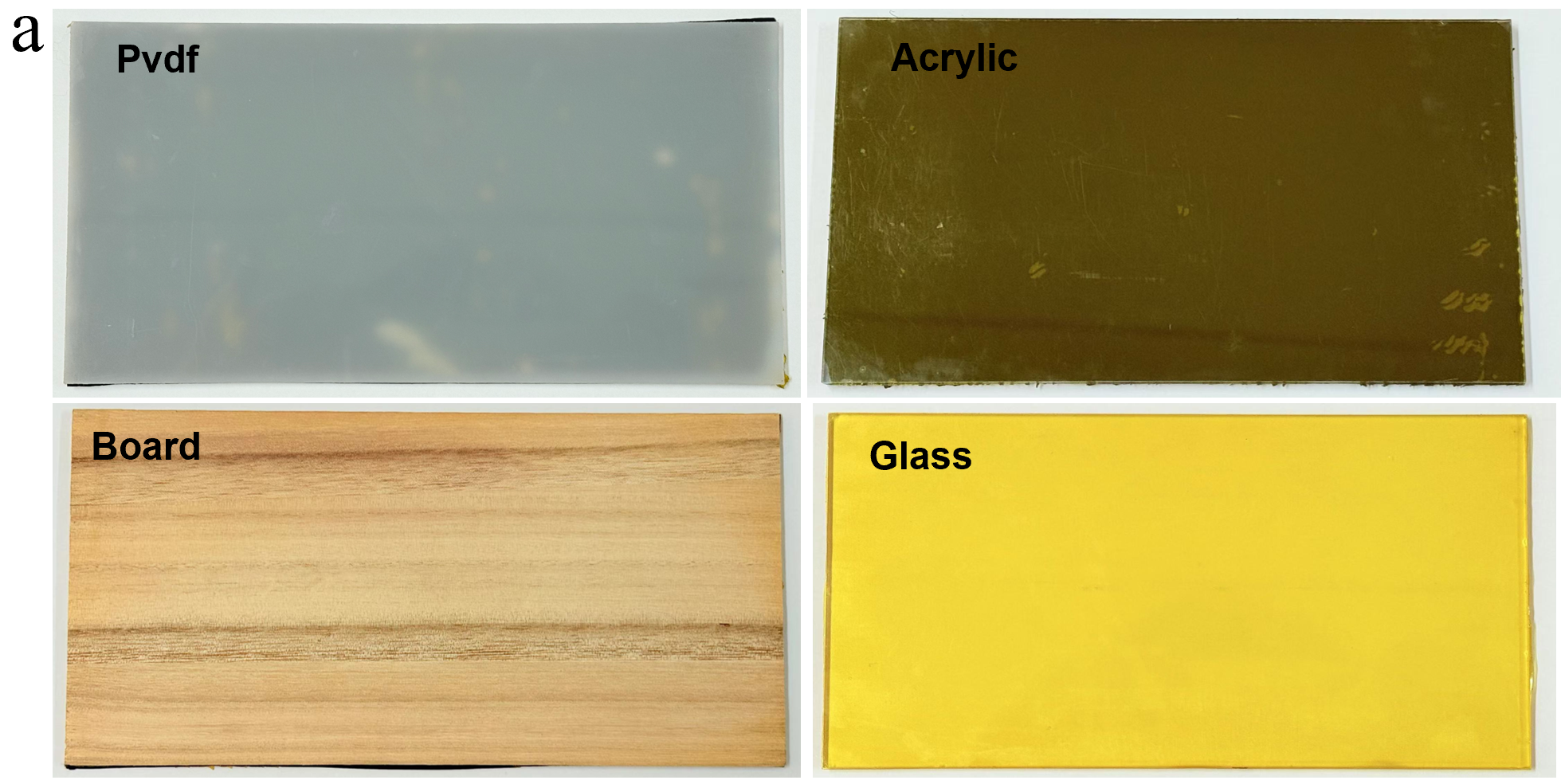
**


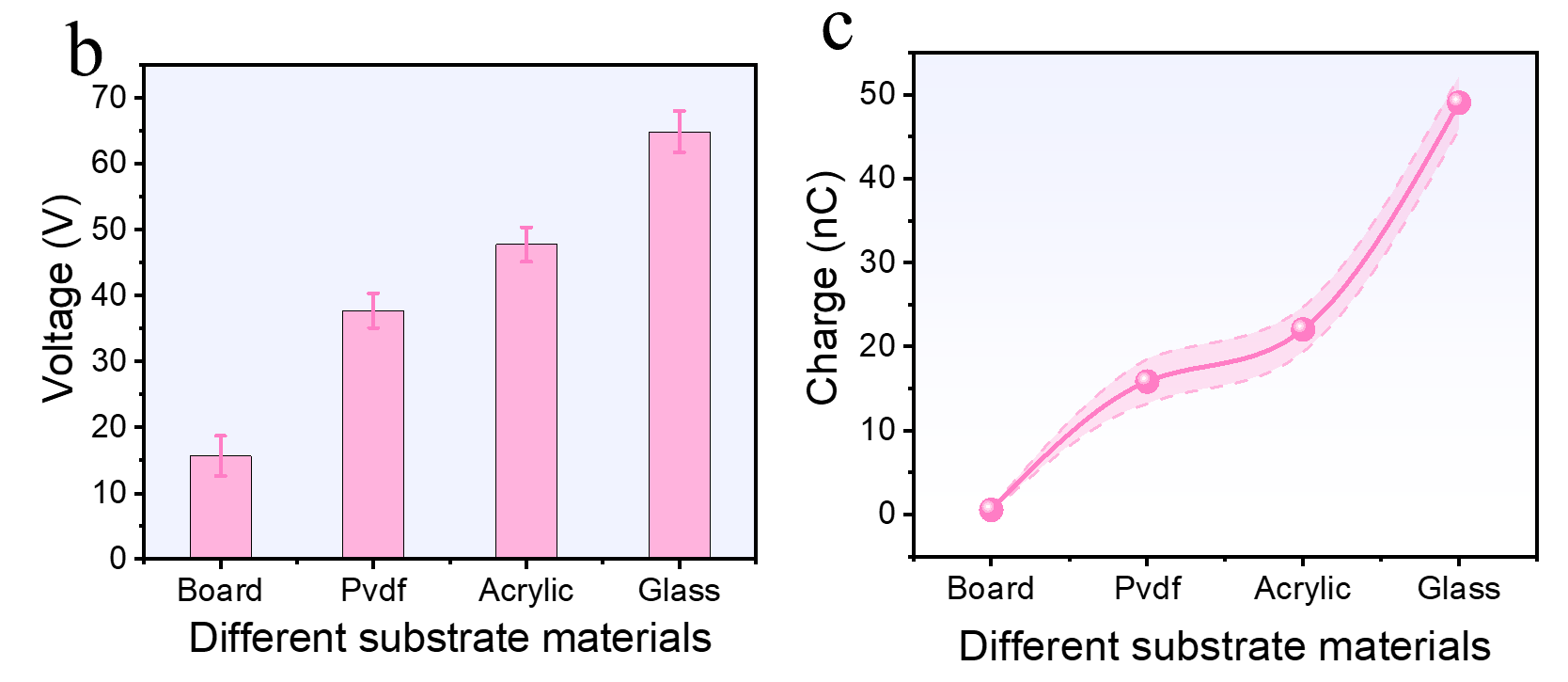


**Figure S14. (a)Four different substrate materials (b)The voltage values(c) The output charge of TENG with different substrate materials.**

**
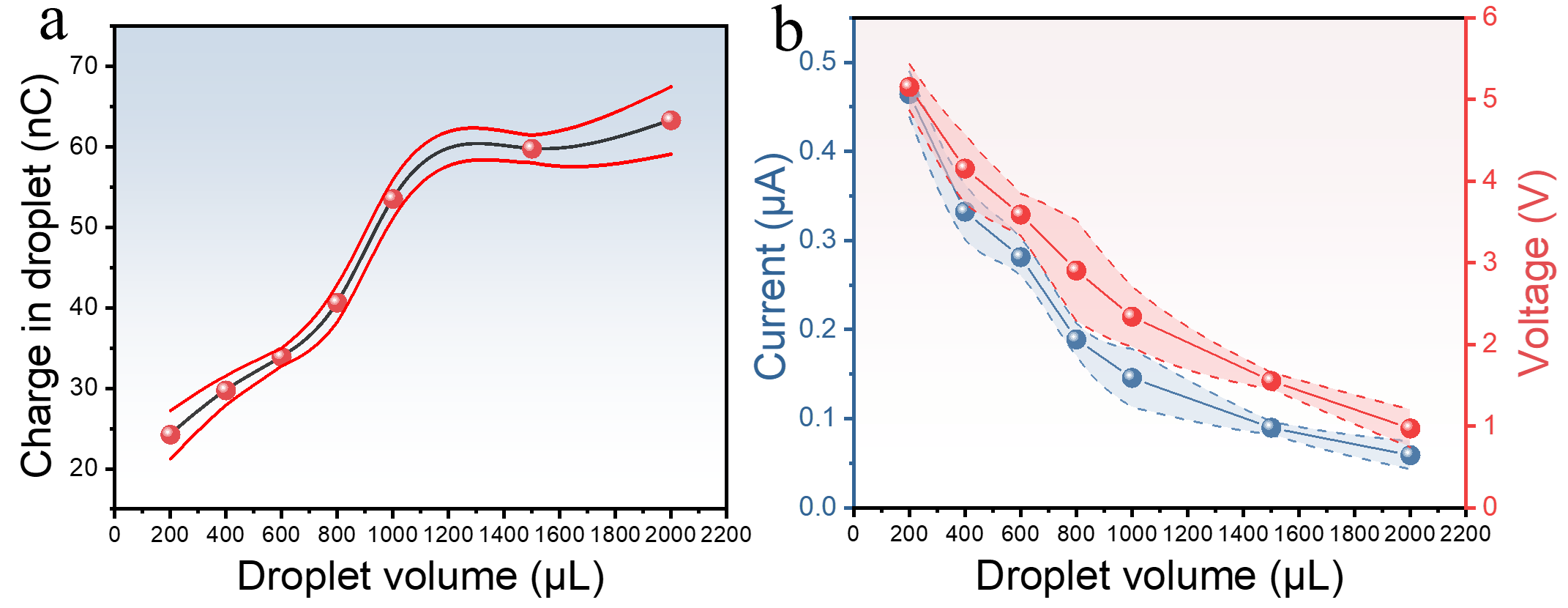
**

**Figure S15. (a)Relationship between droplet volume and injected charge. (b) Correlation between droplet volume and electrical output performance (voltage/current).** As droplet volume increases, surface area and capacitance rise, allowing more charge injection. However, the larger volume reduces the charge injection rate per unit time, increasing internal impedance and lowering current. It also strengthens the reverse internal electric field, weakening the external potential difference. The larger the droplet, the more pronounced this cancellation effect, resulting in a lower open-circuit voltage**.**

**
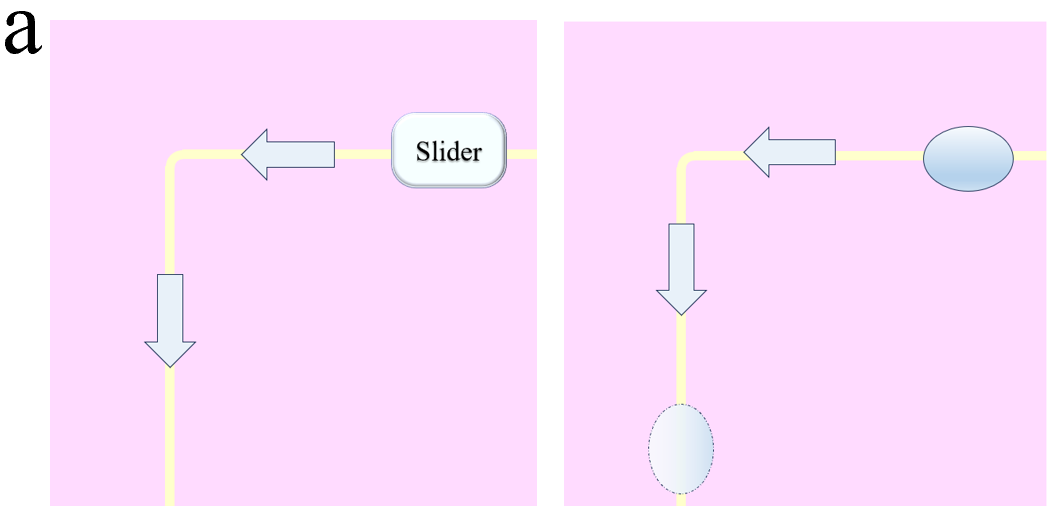
**


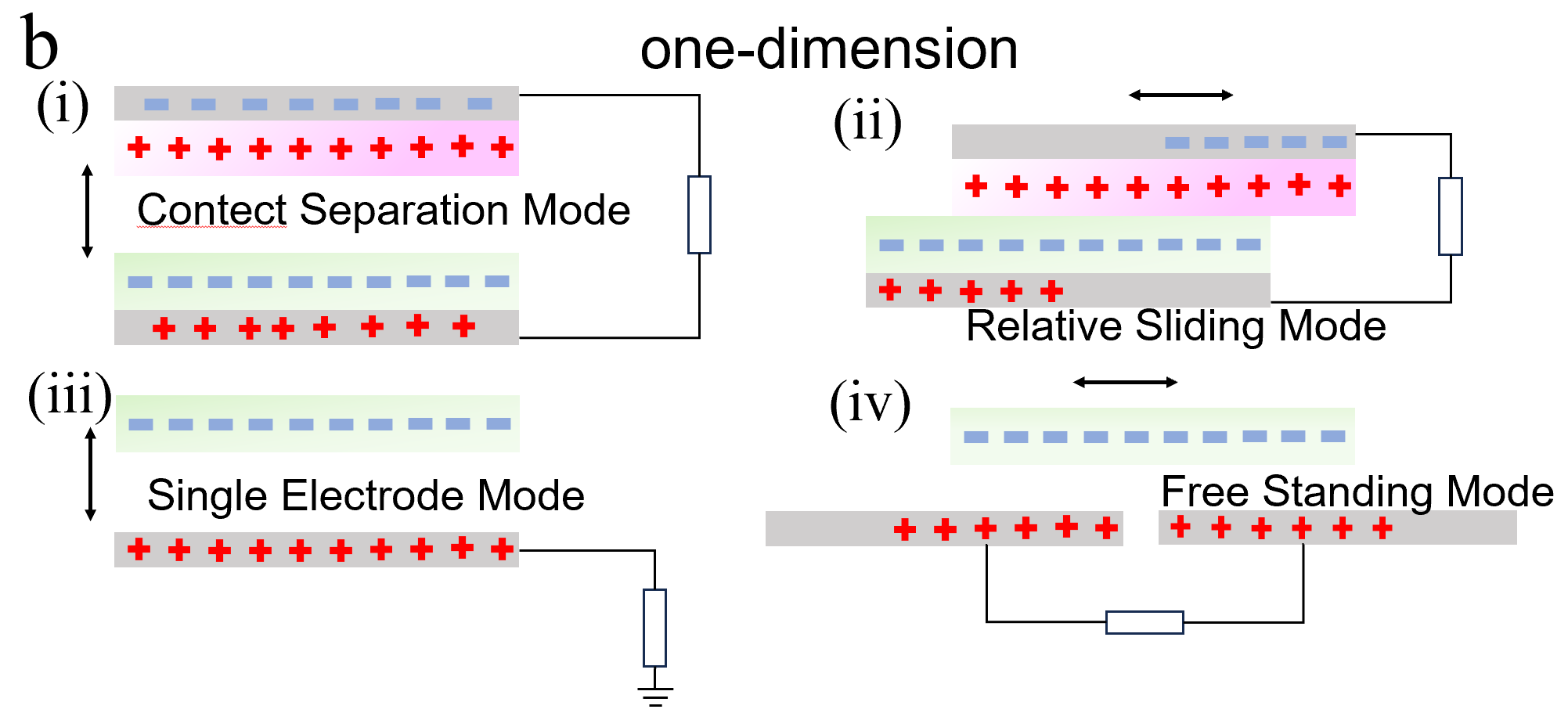


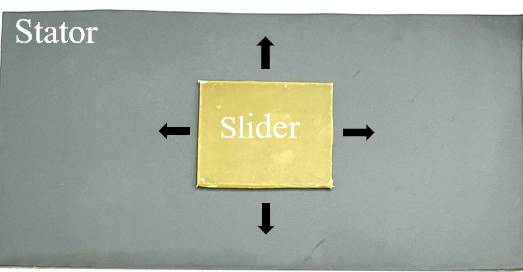


**Figure S16 (a) The motion trajectories of the slider and the droplet.** (b)**The operating mode of DRP-TENG is upgraded from one-dimensional linear motion to two-dimensional planar motion.** TENG is divided into four basic output modes, contact separation (CS-), single-electrode mode (SE-), lateral sliding mode (LS-), and sliding independent friction layer (SFT-). However, the DRP-TENG can work in a two-dimensional plane.


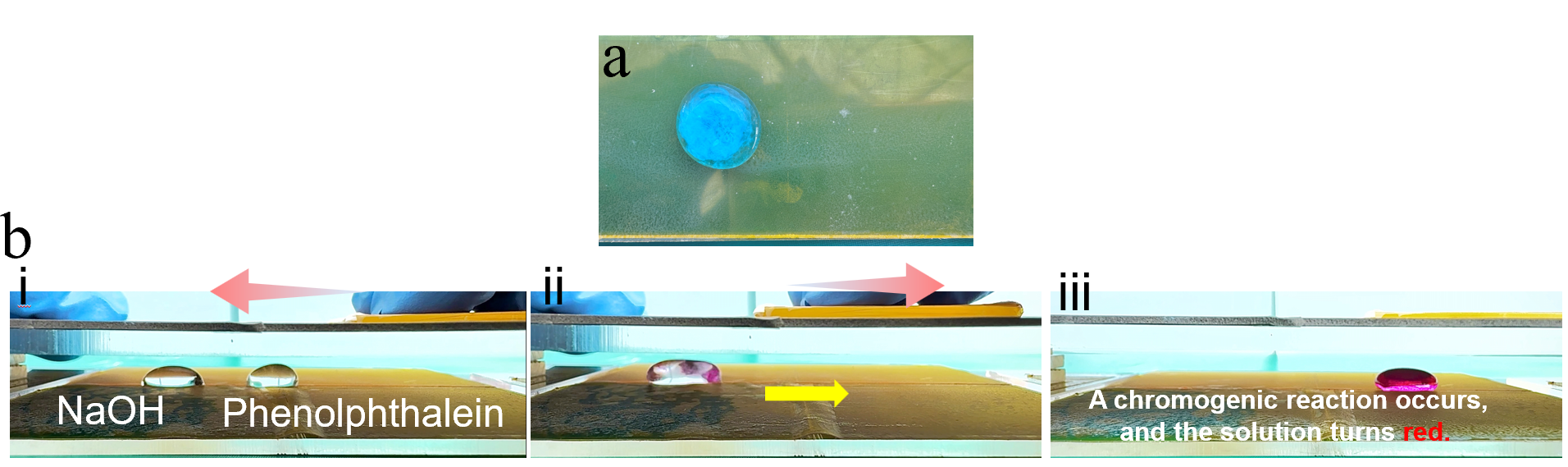


**Figure. S17. (a)** **Photograph of Cu (OH)_2_↓precipitation. (b) The color reaction between NaOH and phenolphthalein droplets, which can continue to proceed after merging.**

**
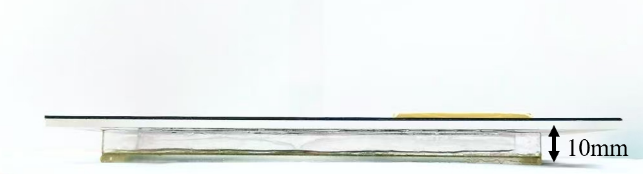

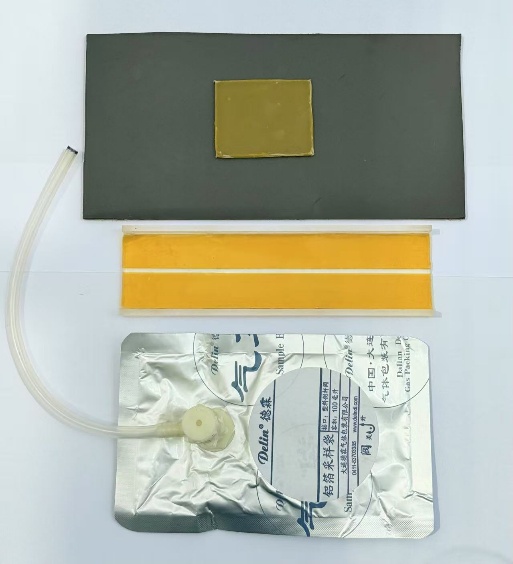
**

**Figure S18.** **Diagram of the experimental device for ammonia monitoring of droplets.**

**
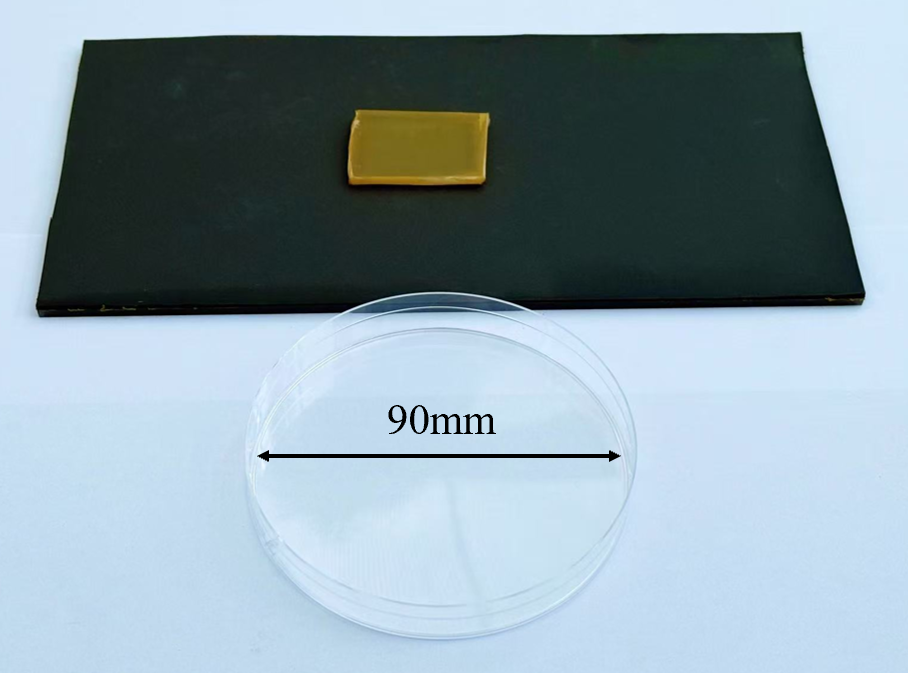
**

**Figure S19.** **Experimental setup of the oil chamber.** The oil chamber is made of acrylic plates with a hydrophobic layer. Its diameter is 90 mm, and 10 mm in height.

**
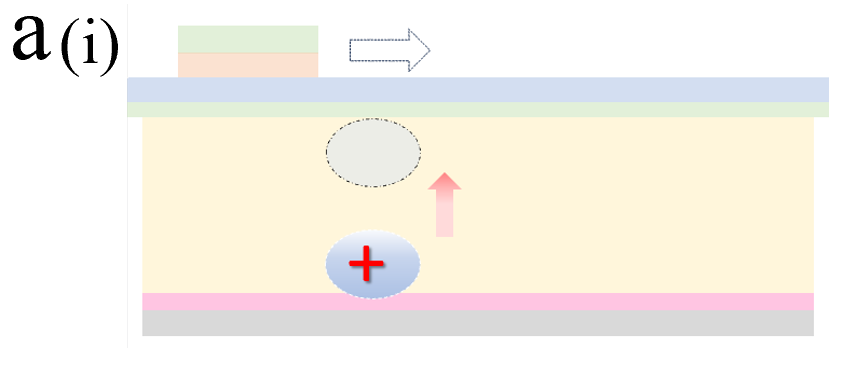

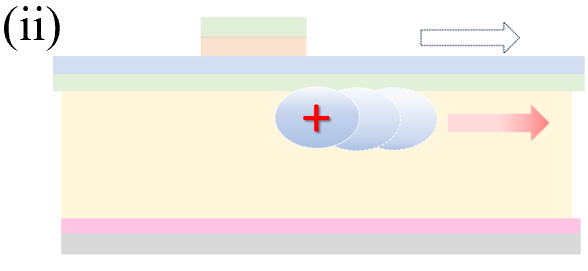

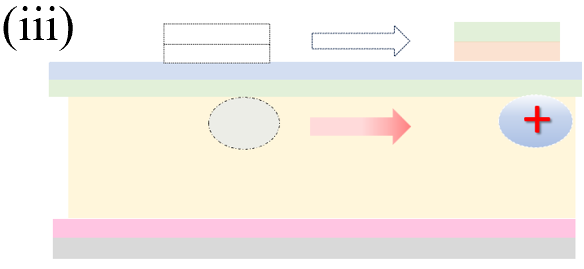
**

**
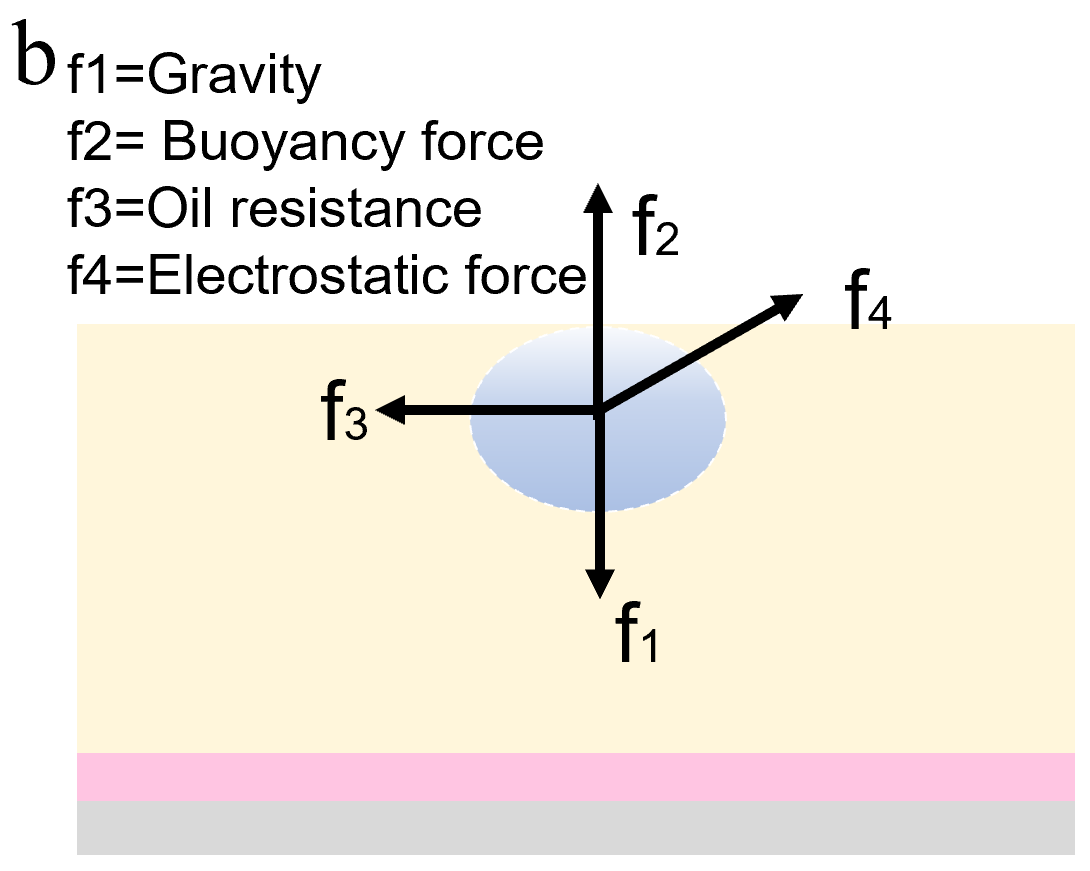
**

**Figure S20. Schematic representation of WEDMS manipulation of droplets within oil layers and Force analysis related to WEDMS manipulation of droplets in oil layers.** (i)The droplet is injected with positive charges and initiates the droplet to float upward. (ii-iii) Manipulate the movement of the suspended droplet.

**
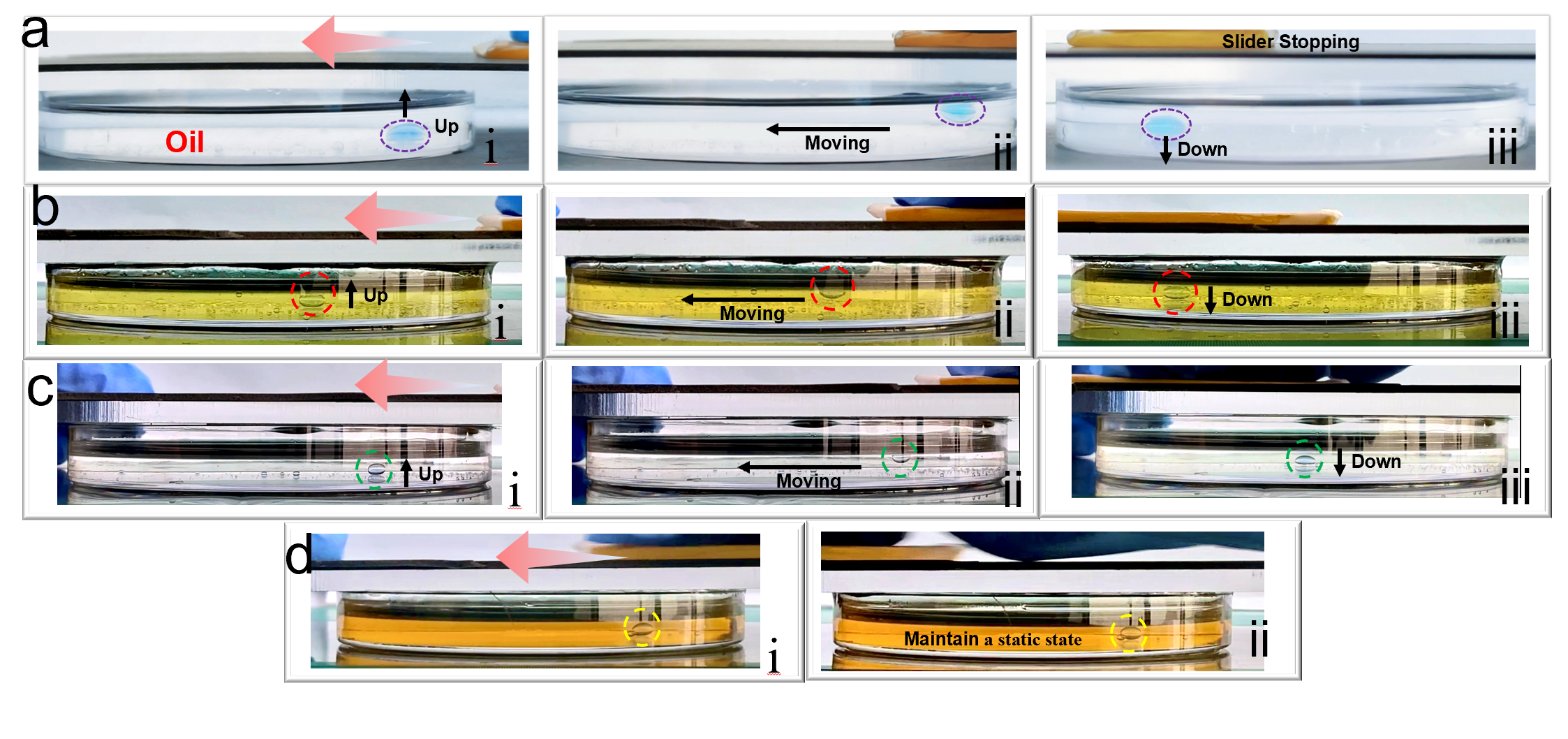
**

**Figure S21.** **Motion states of droplets in four different oils. (a) Dimethylsilicone oil; (b) Vegetable oil; (c) Mineral oil; (d) Engine oil.**

1. **Dimethylsilicone oil:** Its relatively high dielectric constant allows it to generate a fast and controllable response under the EPR-TENG electric field. The droplet moves smoothly and steadily, maintaining a clear trajectory and preserving its shape, demonstrating the most regular and stable motion among the four oil-phase systems.
2. **Vegetable oil:** The droplets are driven by the electric field due to their moderate dielectric constant and viscosity. They accelerate moderately, move at moderate speeds with slight deformation, and generally maintain their spherical or ellipsoidal shape. Although they slide smoothly, their driving distance is shorter than that of dimethylsilicone oil.
3. **Mineral oil:** Droplets respond and accelerate quickly due to their moderate dielectric constant, but higher viscosity leads to oil-phase resistance and deformation, resulting in the fastest fall, shortest driving distance, and least stable motion.
4. **Engine oil:** The moderately high viscosity significantly hinders droplet motion. Under the electric field, droplets exhibit slight surface deformation when the slider passes over them but remain essentially stationary.


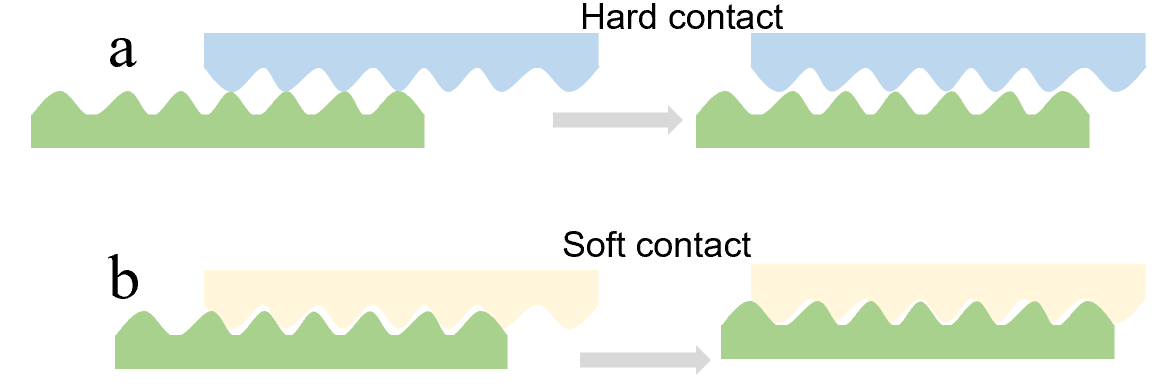


**Figure S22. Hard and soft contact of friction materials.** (a) Microscopic demonstration of hard contact of tribo-materials. (b) Microscopic demonstration of soft contact of tribo-materials.


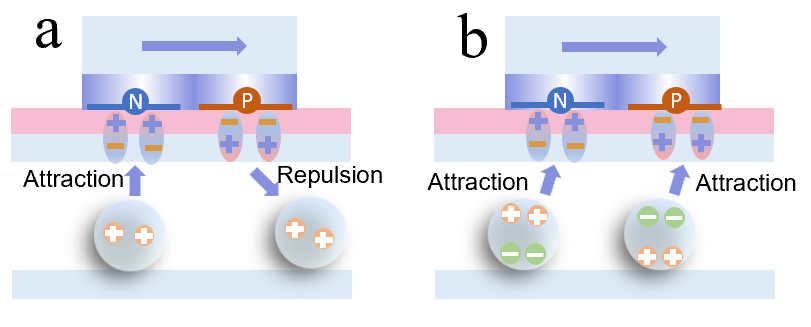


**
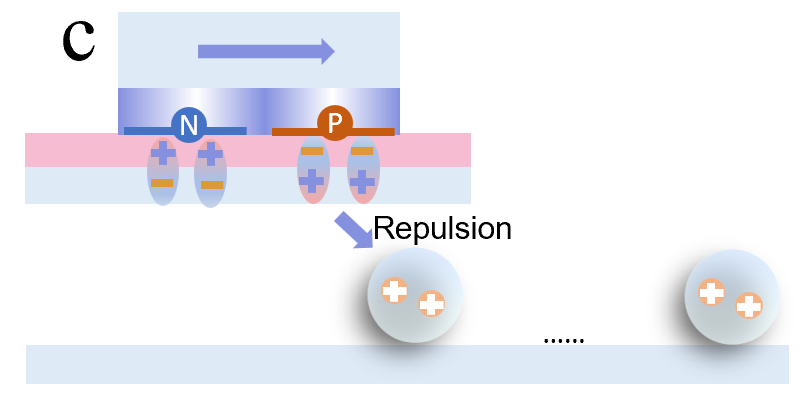
**

**Figure S23. Sources of charge in droplet.** (a) Injected charge in droplet.

(b) Polarization Charge and triboelectric charge in droplet. (c) In this study, when the droplet is placed about 10 mm below the slider, and the slider moves to the right, the TENG generates a strong electric field that injects positive charge into the droplet. Due to the electrostatic repulsion, the droplet moves to the right with a positive acceleration, moving away from the slider.


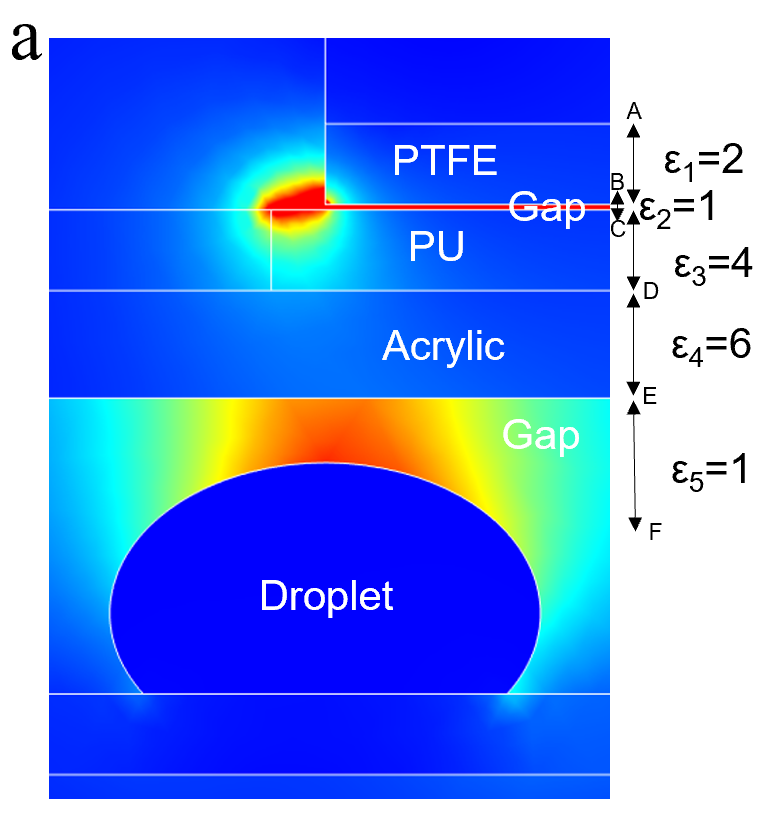

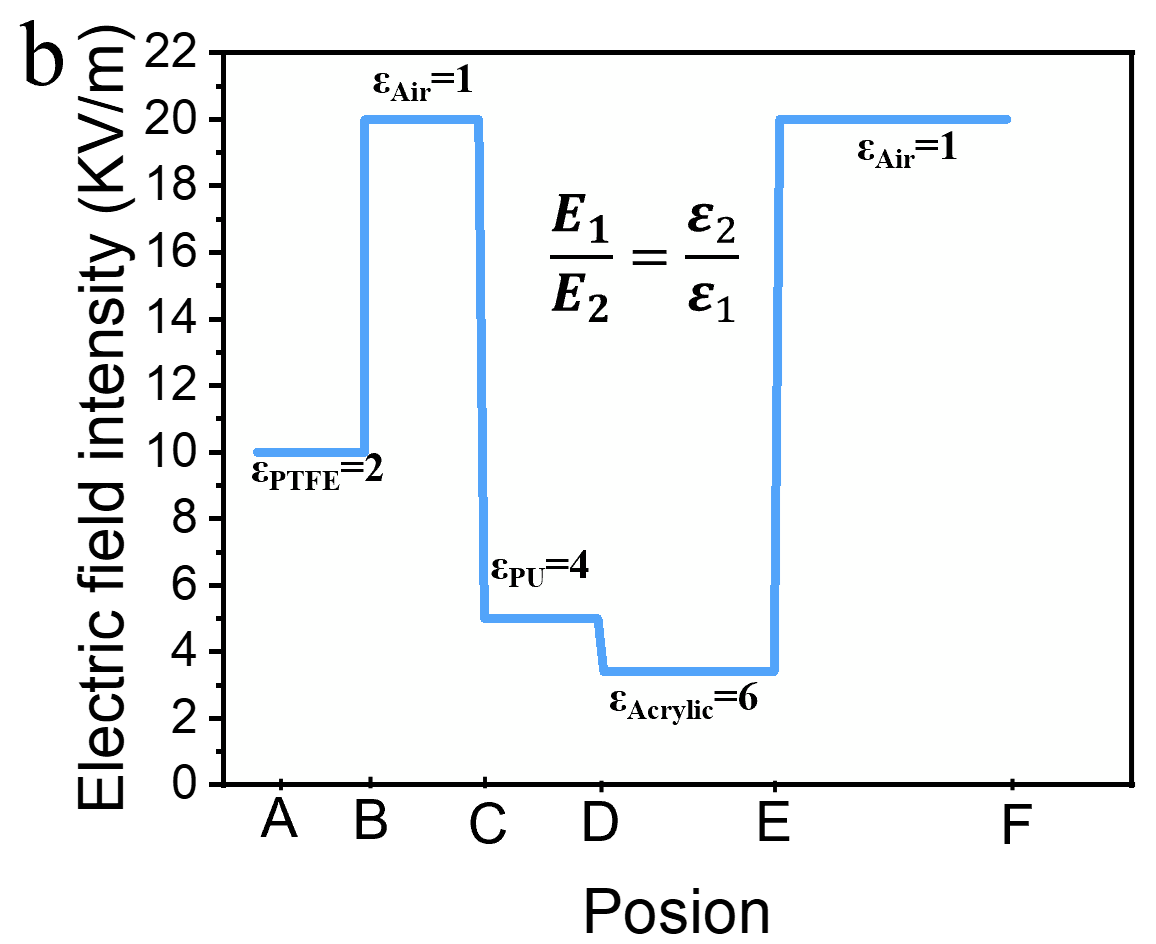


**Figure S24.** **Simulated electric field intensity inside the materials during the WEDMS process.**

**Supplementary Note 1. The soft contact increases the effective area of the interface, which significantly improves the output performance of the DRP-TENG.**

In terms of the selection of soft and hard friction materials, as shown in **Figure S22**, in the case of hard contact, there is a significant gap between the two rigid materials during friction, resulting in insufficient contact between them. Under normal conditions, due to the different electron-binding capacities of the two friction materials, electron transfer occurs between the friction layers. The generated charges are adsorbed on the surfaces of the two friction layers due to electrostatic attraction. When the air gap between the friction layers is too large, the actual contact area between them is significantly reduced, and the system may even operate under "non-contact friction" (e.g., collisions between air molecules). The generated charges may exhibit a phenomenon of "charge dissipation reduction" due to charge escape and other factors, reducing the opportunities for electron transfer. The core reason for this is that charges are less likely to accumulate effectively on the surfaces of the friction layers and are more easily transferred to the surrounding environment, thereby affecting the output performance of the TENG.

In this experiment, a 1mm thick PU foam friction layer was used. Its surface is not completely smooth, as shown in the SEM image in **Figure S3**, presenting a microporous structure and possessing a certain degree of softness. When in contact with the PTFE film, it can fully unfold its potential contact area. The flexible contact better adapts to the micro-irregularities of the friction interface, reducing the "voids" during the contact process. Compared to hard contact, the soft contact has a buffering effect, and the widespread contact points facilitate the effective accumulation of surface charges on the friction layer. The interaction between polyurethane foam and PTFE film is characterized by soft contact, while the interaction between the polyurethane film and PTFE film is characterized by hard contact. Therefore, the closer soft contact between the PU foam and PTFE film results in greater energy output.

**Supplementary Note 2. In the WEDMS system, the injected charge plays a dominant role in droplet manipulation.**

Under the influence of a local electric field discharge, the droplet acquires positive triboelectric charges. Two hypotheses are proposed regarding the interaction force between the droplet and the slider, as illustrated in **Figure S23 (ab)**: (1) Electrostatic charge induces droplet motion (direct charge injection); (2) Droplet polarization induces droplet movement. In the first hypothesis, when the droplet contacts other solid surfaces, electrostatic charges are acquired due to the contact electrification effect (which, for deionized water, is typically positive). As a result, an electric field generated between different friction layers may produce attractive or repulsive electrostatic forces on the droplet. In the second hypothesis, the WEDMS generates a non-uniform electrostatic field, polarizing the droplet into a dipole, which is then attracted by the slider. As shown in **Figure S23 (c)**, the different electric potentials on the left and right sides of the slider always attract the polarized droplet during motion. However, experiments reveal that when the slider moves to the right toward the droplet, the droplet accelerates positively and rapidly moves to the right, away from the slider. Therefore, in the WEDMS system, the motion of the droplet is primarily driven by induced charge.

**Supplementary Note 3. The DRP-TENG slider presents a dual-region opposite-polarization to drive two droplets at the same time.**

Through the combined effects of dielectric polarization and air discharge, DRP-TENG generates a localized electric field, injecting charges into the droplets. Tests with electrodes placed below both sides of the slider revealed that during unidirectional rightward sliding of the PTFE slider, opposite signal phases appeared on both sides. When driving a droplet, the left side of the PTFE friction layer exhibited a positive potential, while the right side exhibited a negative potential. As the sliding distance increased, charges accumulated rapidly. When the electric field strength exceeded the air breakdown threshold, continuous corona discharge occurred on the right side of the friction layer, exerting a repulsive Coulomb force on the positively charged droplet. At this point, the droplet was driven synchronously to the right along with the slider. When the droplet completed unidirectional motion and the slider moved in the reverse direction from left to right, the interface potential distribution switched accordingly, with the left side of the PTFE layer becoming negatively charged and the right side positively charged. As shown in **Figure S8**, when two droplets were placed below the PTFE slider, during rightward sliding, the right side of the slider exerted a repulsive Coulomb force on the droplets, while the left side exerted an attractive Coulomb force, thereby driving both droplets simultaneously. This also indirectly proves the dual-region opposite-polarization potential distribution of DRP-TENG.

**Supplementary Note 4.** **The breakdown voltage of the WEDMS system is calculated according to Paschen's law.**

We estimated the electric field strength based on the measured surface potential and the characteristic distance between the tribo-layer and the droplet. The peak electric field intensity is calculated to be approximately 2.775 kV mm⁻¹, which is below the breakdown threshold of air under ambient conditions (~3 kV mm⁻¹) but sufficient to induce charge injection and motion of the droplet. In fact, corona discharge phenomena were occasionally observed under high excitation conditions. The captured corona images provide experimental evidence for the existence of strong localized electric fields near the triboelectric interface, as shown in **Fig.1D (**in the manuscript**)**. These results indicate that the electric field intensity can approach the ionization threshold of air. The electric field intensity can be estimated as E = $\frac{V}{d}$, the actual breakdown voltage measured was 8327 V. The calculated electric field strength is below the breakdown threshold of 3 kV/mm, yet it is still sufficient to cause air breakdown and drive droplet motion. This phenomenon can be attributed to the highly localized surface charge distribution generated by the triboelectric effect. Unlike the uniform electric field produced by a high-voltage power supply, the TENG generates a strong non-uniform electric field with large field gradients near the surface. Since the electrostatic force acting on the droplet is proportional to the gradient of the electric field, this localized field enhancement can result in an instantaneous peak that may exceed the threshold, and is sufficient to induce droplet oscillation and movement.

Based on this, we calculated the breakdown voltage of the system using the theoretical Paschen’s law, and the results are presented below:

$$\begin{aligned} V_{b}=A\frac{Pd}{\left( \ln\left( Pd \right)+B \right)}\#\left( 1 \right) \end{aligned}$$

**Where *A*=273.75(V/Pa·m),*B*=1.08，The atmospheric pressure of the laboratory -*P*=7.4158×10^4^(Pa)，Distance between the electrode and the droplet-*d*=3(mm).**

$$\begin{aligned} V_{b}=A\frac{Pd}{\left( \ln\left( Pd \right)+B \right)}\approx9378.39(V)\#\left( 2 \right) \end{aligned}$$

The breakdown voltage obtained from theoretical calculations is higher than **the experimentally measured value (8237 V)**, which can be attributed to two factors. First, the use of copper wire electrodes introduces tip effects that locally amplify the electric field, causing dielectric breakdown at a voltage lower than the theoretical prediction. Second, the relatively high humidity in Kunming allows water vapor to adsorb at the electrode-dielectric interface, forming weak conductive paths that reduce the effective electric field at the interface, further lowering the measured breakdown voltage.

**Supplementary Note 5. The distribution of electric field intensity across different materials during discharges facilitated by dielectric polarization.**

To further investigate the physical mechanisms behind dielectric polarization discharge in the DRP-TENG friction layer, we simulated the model using COMSOL software, as shown in **Figure S24(a)**. The electric field intensity distribution curve from point A to point F within the structure is presented in **Figure S24(b)**. The data indicate that, when neglecting the penetration of the dielectric, the ratio of the electric field intensities for different materials is exactly the reciprocal of their relative dielectric constants.

which can be expressed as

$$\begin{aligned} \frac{E_{1}}{E_{2}}=\frac{\varepsilon_{2}}{\varepsilon_{1}}\#\left( 1 \right) \end{aligned}$$

Where *E_1_/E_2_*​ and *ε_1_/ε_2_*​, represent the electric field intensities inside dielectric 1 and dielectric 2, and the relative dielectric constants of dielectric 1 and dielectric 2, respectively. The results are consistent with the friction layer dielectric polarization model. In this process, frictional charges can spontaneously polarize the dielectric polymer and form an electric field, the electric dipoles induced at the bottom of the friction layer can be regarded as binding surface charges. This phenomenon is referred to the dielectric polarization enables discharge, where dielectric polymers function similarly to charge transfer bridges.

**Supplementary Note 6. Force analysis related to WEDMS manipu-lation of droplets in oil layers.**

The forces acting on the droplet within a thin oil layer are analyzed in **Figure S20(b)**，where the droplet experiences gravity (f1), buoyancy force(f2), oil resistance (f3) and electrostatic force (f4).(g=9.8m/s², ρ_0_=0.974 g/cm^3^, k=9×10^9^ N⋅m2/C2,η=500 mm^2^/s,C_d_​=$\frac{24}{Re}$,Re=$\frac{v\times2r}{\eta}.$)

$$\begin{aligned} f_{2}=p_{0}Vg\#\left( 2 \right) \end{aligned}$$

$$\begin{aligned} f_{3}=\frac{1}{2}C_{d}\rho_{o}Sv^{2}\#\left( 3 \right) \end{aligned}$$

$$\begin{aligned} f_{4}=k\frac{q_{1}q_{2}}{\left( h \right)^{2}}\#\left( 4 \right) \end{aligned}$$

Where ρ_0_ represents the density of the oil, while *V* denotes the volume of the droplet immersed in the oil. *C* is the oil drag coefficient, *S* refers to the surface area of the droplet in contact with the oil, and v indicates the droplet’s velocity. The vertical force meets the conditions of f_4y_+f_2_ = f_1_, which maintains the dynamic balance of the droplets and ensures the stable suspension of the droplets. The horizontal traction of droplet movement under oil base is f_4x_-f_3_.

**Supplementary Table 1.** The surface tension and the sphericity characteristics exhibited by the droplet samples that can be driven by the WEDMS system.

| **Droplet Category** | **Surface Tension / Polarity** | **Shape** |
| --- | --- | --- |
| **Water** | 72.8 mN/m | The hydrophobic surface has a relatively good spherical shape |
| **Electrolyte solution (salt solution)** | Slightly higher than pure water | Spherical stability, strong conductivity |
| **Dilute alkaline/dilute acidic solution** | 73 ~ 78 mN/m | Spherical Stability |
| **Mercury** | 486 mN/m | Almost perfectly spherical |
| **Liquid metal** | 500–700 mN/m | Extremely spherical |
| **Non-surface-active water-soluble organic solution (glucose, sucrose, PAM)** | High surface tension/high polarity | Spherical stability |
| **Albumin serum, serum** | High surface tension | Spherical stability |

Their common characteristic is that they all have a high surface tension. For effective actuation in this system, the liquid must form spherical droplets on hydrophobic surfaces, which requires relatively high surface tension. Most electrolyte solutions, water-soluble non-surfactant aqueous solutions, and polar small-molecule aqueous solutions, such as acids, bases, salts, PAM, and glucose, have high surface tension, high polarity, and non-surfactant characteristics. These solutions exhibit poor compatibility with hydrophobic interfaces but can maintain stable spherical morphologies. Using charge injection and electrostatic forces, these droplets can be precisely controlled. In contrast, surface-active organic solvents, such as alkanes, benzene, and oils, due to their low surface tension and strong affinity for hydrophobic surfaces, tend to spread and diffuse easily on hydrophobic layers. As a result, they are unable to maintain stable spherical shapes and lose their rolling ability, making them unsuitable for this actuation system.

**Supplementary Table 2**. **Performance comparison of electronically controlled droplets^[1-6]^**

|  | TCCI^[1]^ | OTT^[2]^ | DCI^[3]^ | TEW^[4]^ | TEP^[5]^ | EPD^[6]^ | This work |
| --- | --- | --- | --- | --- | --- | --- | --- |
| Human droplet interaction | **√** | **√** | **×** | **√** | **√** | **×** | **√** |
| No need for wires or electrodes | **×** | **×** | **×** | **√** | **×** | **×** | **√** |
| Oil-phase medium | **×** | **√** | **×** | **×** | **√** | **×** | **√** |
| Control droplet start and stop | **×** | **√** | **×** | **√** | **√** | **√** | **√** |
| Multiple droplets | **×** | **×** | **√** | **×** | **×** | **√** | **√** |
| Closed channel | **×** | **√** | **×** | **√** | **√** | **×** | **√** |
| Large droplets＞1000μL | **√** | **×** | **×** | **×** | **×** | **×** | **√** |

**(1)** **TCCL: Triboelectric contactless charge injection method.**

**(2) OTT: Omni-directional triboelectric tweezer.**

**(3) DCI: Human-motion-induced direct charge injection.**

**(4) TEW: Electrowetting on dielectric.**

**(5) TEP: Tribo-electrophoresis.**

**(6) EPD: Electret-induced polarization on droplet.**

**(7) WEDMS: Omni-directional wireless electro- droplet manipulation system.**

1. **Human droplet interaction：Can physical actuation of microdroplets be achieved through simple human mechanical sliding?**
2. **No need for wires or electrodes：The system operates without relying on any arrayed electrodes or rigid wired circuits.**
3. **Oil-phase medium：Can 3D spatial transport of droplets be achieved in an oil-phase medium?**
4. **Control droplet start and stop：Can the start and stop of droplets be controlled in real time?**
5. **Multiple droplets：Can multiple droplets be manipulated simultaneously?**
6. **Closed channel：Can droplets be manipulated inside a closed channel (space)?**
7. **Large droplets>1000μL：Can droplets larger than 1000 μL in volume be manipulated?**

**Supplemental Movies**

**Movie S1.**

Manipulate the droplets along the curve.

**Movie S2.**

Manipulate droplets for instant start-stop.

**Movie S3.**

WEDMS system Manipulates two droplets.

**Movie S4.**The real-time current output of the DRP-TENG .

**Movie S5.**

Effective drive range of the WEDMS system.

**Movie S6**

Manipulate droplets of different volumes.

**Movie S7.**

Manipulate 2000μL droplet.

**Movie S8.**

The WEDMS system manipulates the charge shuttle of the droplets.

**Movie S9.**

The droplets combine to produce a chemical reaction

**Movie S10**

WEDMS Manipulates Solid-Liquid System

**Movie S11.**

Droplet detect ammonia in a closed space

**Movie S12.**

WEDMS manipulates the movement of droplets in oil

**Supplementary References**

1. Tan, L.M., Zeng, Q., Xu, F., Zhao, Q., Chen, A., Wang, T., Tao, X., Yang, Y., and Wang, X. *Adv. Mater.***2024**, 36, 2313878.
2. Sun, J.F., Zhao, Q., Mo, Z., Chen, J., Guo, H., and Zhang, L. *Nat. Commun.***2025**, 16, 2312.
3. Sun, J.F., Zhang, L.J., Zhou, Y., Li, Z., Libanori, A., Tang, Q., Huang, Y., Hu, C., Guo, H., Peng, Y., and Chen, J. *Materials Today.* **2022**,58, 41-47.
4. Tan, J., Fan, Z., Zhou, M., Liu, T., Sun, S., Chen, G., Song, Y., Wang, Z., and Jiang, D. Adv. Mater. **2024**,36, 2314346.
5. Sun, J.F., Zhang, L., Gong, S., Chen, J., and Guo, H. Adv. Mater. **2023**, 35, 2305578.
6. Zhang, R.T., Zhang, C., Fan, X., Au Yeung, C.C.K., Li, H., Lin, H., and Shum, H.C. *Nat. Commun.* **2024**,15, 6220.
